# *Corynebacterium* Cell Factory Design and Culture Process Optimization for Muconic Acid Biosynthesis

**DOI:** 10.1038/s41598-018-36320-4

**Published:** 2018-12-21

**Authors:** Han-Na Lee, Woo-Shik Shin, Seung-Yeul Seo, Si-Sun Choi, Ji-soo Song, Ji-yeon Kim, Ji-Hoon Park, Dohoon Lee, Sang Yong Kim, Sang Joung Lee, Gie-Taek Chun, Eung-Soo Kim

**Affiliations:** 1 Department of Biological Engineering, Inha University, Incheon, 22212 Republic of Korea; 2 Green Chemistry and Materials Group, Korea Institute of Industrial Technology, Cheonan-si, Chungcheongnam-do 31056 Republic of Korea; 3 STR Biotech Co., Ltd., Bioplaza 4-3, 56, Soyanggang-ro, Chuncheon-si, Gangwon-do, 24232 Republic of Korea; 4 Department of Molecular Bio-science, Kangwon National University, Chuncheon-si, Gangwon-do, 24341 Republic of Korea; 5 Green Process and System Engineering Major, Korea University of Science and Technology (UST), Daejeon, 34141 Republic of Korea

## Abstract

Muconic acid (MA) is a valuable compound for adipic acid production, which is a precursor for the synthesis of various polymers such as plastics, coatings, and nylons. Although MA biosynthesis has been previously reported in several bacteria, the engineered strains were not satisfactory owing to low MA titers. Here, we generated an engineered *Corynebacterium* cell factory to produce a high titer of MA through 3-dehydroshikimate (DHS) conversion to MA, with heterologous expression of foreign protocatechuate (PCA) decarboxylase genes. To accumulate key intermediates in the MA biosynthetic pathway, *aroE* (shikimate dehydrogenase gene), *pcaG/H* (PCA dioxygenase alpha/beta subunit genes) and *catB* (chloromuconate cycloisomerase gene) were disrupted. To accomplish the conversion of PCA to catechol (CA), a step that is absent in *Corynebacterium*, a codon-optimized heterologous PCA decarboxylase gene was expressed as a single operon under the strong promoter in a *aroE-pcaG/H-catB* triple knock-out *Corynebacterium* strain. This redesigned *Corynebacterium*, grown in an optimized medium, produced about 38 g/L MA and 54 g/L MA in 7-L and 50-L fed-batch fermentations, respectively. These results show highest levels of MA production demonstrated in *Corynebacterium*, suggesting that the rational cell factory design of MA biosynthesis could be an alternative way to complement petrochemical-based chemical processes.

## Introduction

*Cis*, *cis*-muconic acid (MA), a C6 unsaturated dicarboxylic acid, is a precursor and platform chemical for the production of commercially important bulk chemicals such as adipic acid and terephthalic acid^1,2^. Adipic acid and terephthalic acid are chemicals used in the production of various types of plastics including polyethylene terephthalate (PET) and nylon-6,6. Currently, the main method for the production of adipic acid is by chemical syntheses using petroleum-based feedstock including benzene and high-concentration heavy metals^3,4^, or as a combination of two isomers of *cis, cis-*MA and *cis, trans*-MA, which requires expensive catechol (CA)^5^. Because the intermediate products produced by chemical reactions are toxic and cause environmental problems, it is necessary to develop environmentally friendly and economical MA production using engineered microorganisms and low-cost materials^4^.

**Figure 1:**
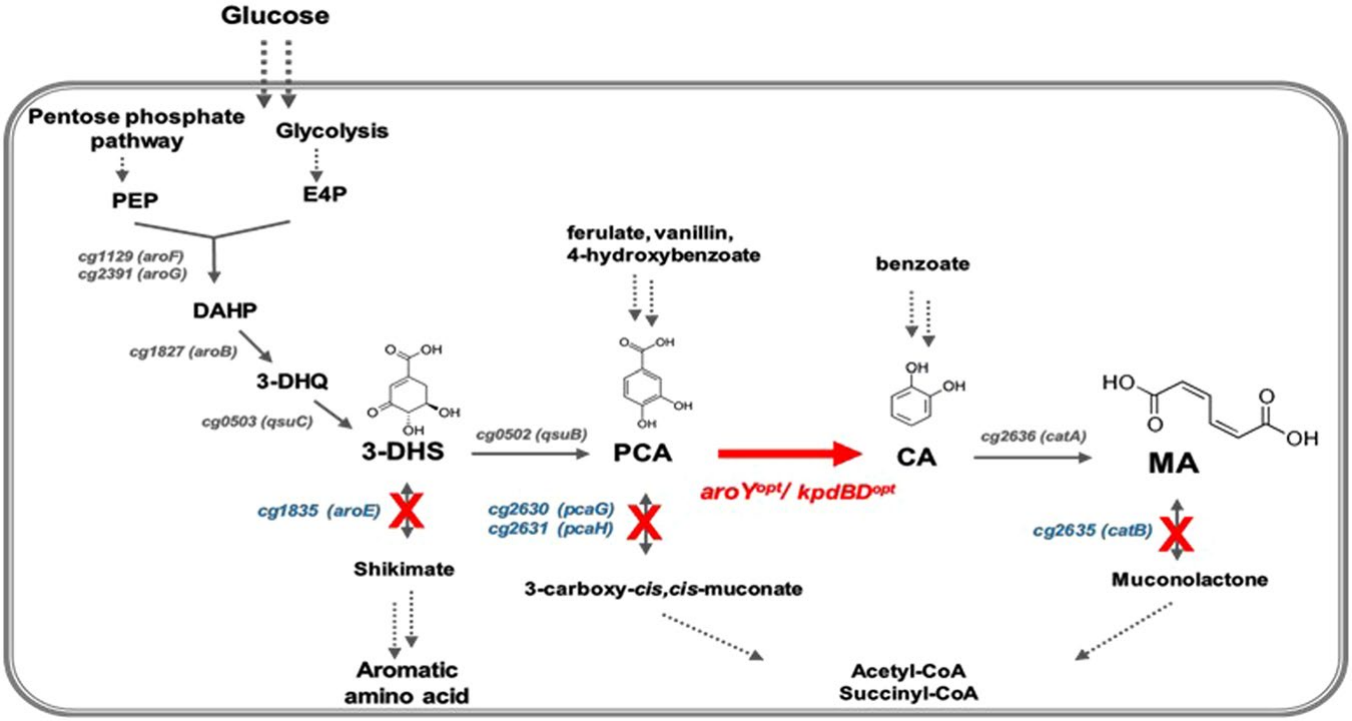
Metabolic pathway for muconic acid production in *Corynebacterium glutamicum*. The red lines represent the engineered path, and the blue text represents disrupted genes. Dashed arrows indicate multiple enzyme reactions. PEP, phosphoenolpyruvate; E4P, erythrose 4-phophate; DAHP, 3-deoxy-D-arabino-heptulosonate-7-phosphate; 3-DHQ, 3-dehydroquinate; 3-DHS, 3-dehydroshikimate; PCA, protocatechuate; CA, catechol, MA, muconic acid. Genes and coded enzymes: *aroF* and *aroG*, phospho-2-dehydro-3-deoxyheptonate aldolase; *aroB*, DHQ synthase; *qsuC*, shikimate/quinate dehydratase; *aroE*, shikimate 5-dehydrogenase; *qsuB*, phosphate isomerase; *pcaG/H*, PCA deoxygenase α/β subunits; *aroY*^*opt*^/*kpdBD*^*opt*^, codon-optimized PCA decarboxylase and subunits; *catA*, CA 1,2-dioxygenase; *catB*, chloromuconate cycloisomerase.

MA can be biosynthesized through the redesign of aromatic amino acid biosynthetic pathways that can be found in most microorganisms, or the degradation pathway of benzene compounds, found in some microorganisms^6–10^. Several studies on microorganism-based MA biosynthesis have been reported^1,2,11–13^. Frost and Draths successfully biosynthesized MA from glucose for the first time using *E. coli*^14^. They introduced three foreign genes (*aroZ, aroY*, and *catA*) into the aromatic amino acid biosynthetic pathway, to produce MA *via* stepwise conversions in the shikimate pathway (Fig. 1). According to their report, the main target of MA biosynthesis was DHS, the intermediate of the shikimate pathway. DHS dehydratase (AroZ derived from *Klebsiella pneumoniae*) catalyzed DHS to PCA, PCA decarboxylase (AroY derived from *Klebsiella pneumoniae*) converted PCA to CA, and catechol 1,2-dioxygenase (CatA derived from *Acinetobacter calcoaceticus*) transformed CA to MA. Subsequently, the engineered *E. coli* strains with enhanced production of phosphoenolpyruvate (PEP) and erythrose 4-phosphate (E4P), which were precursors of MA biosynthesis, were constructed to produce MA with a final titer of 36.8 g/L in the fed-batch fermentation^15^. Studies on the production of MA from various types of carbon sources using *E. coli, Saccharomyces cerevisiae, Pseudomonas*, and *Amycolatopsis* strains have been reported^1,16–19^. In addition, more studies have reported production of MA using a different pathway, from chorismate or anthranilate, though it has low productivity and requires improvement^20–22^.

*Corynebacterium glutamicum*, which is a gram-positive soil bacterium that can produce amino acids such as L-glutamate and L-lysine on an industrial scale, has recently seen wide use in biorefinery fields that produce chemicals from lignocellulosic biomass or mixed sugars containing glucose^23–25^. Several studies on metabolic engineering to produce shikimate from *C. glutamicum* as a raw material have also been reported^26–28^. *C. glutamicum*, whose genome is well characterized, has the ability to produce amino acids on a large-scale, indicating that it could be an ideal host for MA production using the aromatic amino acid biosynthetic pathway. In a previous study, *Corynebacterium* was used to produce 2−3 g/L MA from benzoate in batch or fed-batch fermentation^29,30^.

In this study, we engineered a *C*. *glutamicum* strain to produce MA from glucose through redesign of the aromatic amino acid biosynthetic pathway. Through heterologous expression of a codon-optimized PCA decarboxylase gene cluster under the strong promoter, as well as deletions of several key genes involved in MA intermediate bypass routes, an optimized pathway for DHS-PCA-CA-MA was successfully constructed in *C. glutamicum. C. glutamicum* is a well-known cell factory microorganism suitable for metabolic engineering and has long been used to produce various aromatic amino acids in a large-scale fermentation process. *C. glutamicum* requires only single heterologous gene to convert PCA to CA, rather than three heterologous genes required in *E. coli*, which is advantageous in designing metabolic circuits for MA production. Moreover, optimization of culture media and processes using the MA-producing *C. glutamicum* strain were performed to achieve a significantly increased titer of MA, suggesting that the rational cell factory design in *Corynebacterium* could be an efficient method for MA production.

## Results

### Redesign of the *Corynebacterium* shikimate pathway

To establish an MA biosynthetic pathway similar to that previously described for *E. coli*, we engineered a *Corynebacterium* shikimate pathway. The *aroE*-encoding shikimate dehydrogenase was deleted from the chromosome to block the conversion of DHS, causing DHS to accumulate in the cells.

There were three shikimate/quinate dehydrogenase genes (cg0504, cg1283 and cg1835) in *C. glutamicum* ATCC13032 (http://www.coryneregnet.de). Among them, the cg1835 gene showed 98% identity with *aroE* (cgR1677), which is known as the main shikimate dehydrogenase in the *C. glutamicum* R strain^31^. We decided to delete the cg1835 gene in wild-type *C. glutamicum*, yielding a strain named STR001. The STR001 strain, whose *aroE* gene was deleted, could not fully grow on LB and BT agar medium, and could grow in minimal media only in the presence of aromatic amino acids (Supplementary Fig. 1). Although DHS never accumulated in the wild type, it was confirmed to be accumulated in the STR001 strain, using HPLC analysis (Supplementary Fig. 2).

No PCA accumulation was observed, even though the DHS dehydratase-coding gene (*qsuB*, cg0502) involved in the conversion of DHS to PCA were present in *C. glutamicum*. Accordingly, it was speculated that PCA could be degraded by *pcaG/H* to form β-carboxy *cis, cis*-muconate, and further metabolized to acetyl-CoA or succinyl-CoA through a β-ketoadipate intermediate (Fig. 1). To block the conversion of PCA to β-carboxy-*cis,cis*-muconate, we decided to generate the strain that could accumulate PCA in the cells. The PCA 3,4-dioxygenase was composed of PcaG (cg2630) and PcaH (cg2631), representing the α and β subunits, respectively. The *pcaG/H* genes were additionally deleted in the STR001 strain, and the resulting strain was named as STR002. As expected, DHS and PCA were confirmed to be accumulated in the STR002, using HPLC analysis (Supplementary Fig. 1).

To prevent the metabolic processing of MA to muconolactone, and thus obtaining MA as the final product, it was necessary to delete *catB*, which encodes chloromuconate cycloisomerase (Fig. 1). The *catB* gene was also deleted using a similar scheme as for other genes, yet no MA was accumulated in the resulting STR003 strain (Supplementary Fig. 1). To confirm that in principle MA accumulation is possible^32^, benzoate was added to strain STR003. As expected, MA was confirmed to be accumulated when benzoate was supplemented in the culture medium (Supplementary Fig. 3, Supplementary Table 2). Even when the catechol was added to the medium, a small amount of MA was also detected, suggesting that the conversion from benzoate/catechol to MA was possible.

### Functional heterologous expression of PCA decarboxylase genes

**Figure 2:**
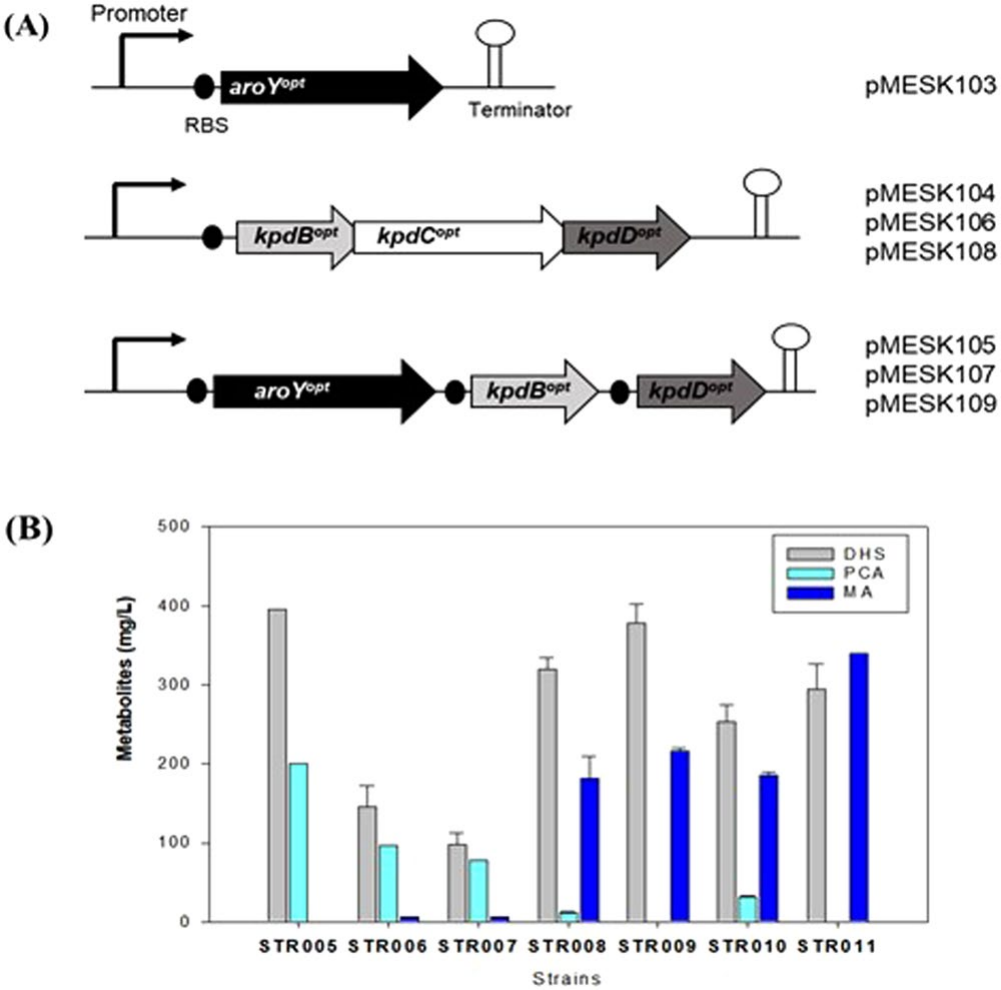
(**A**) Schematic representation of the operons, including the PCA decarboxylase gene and/or subunit genes, constructed in this study. *aroY*^*opt*^ or *kpdC*^*opt*^, codon-optimized decarboxylase gene derived from *K. pneumoniae; kpdB*^*opt*^ and *kpdD*^*opt*^, codon-optimized PCA decarboxylase subunit genes derived from *K. pneumoniae. kpdB*^*opt*^, *kpdC*^*opt*^ and *kpdD*^*opt*^ genes are transcriptionally coupled. RBS represents ribosome binding site. (**B**) Production of metabolites by *C. glutamicum* strains harboring gene operons (presented in panel A). *Grey*, DHS; *sky blue*, PCA; *blue*, MA. Error bars represent standard deviations based on duplicate experiments.

Since we confirmed that MA could be synthesized from benzoate *via* the β-ketoadipate pathway as described above, we expected that MA could be produced by linking the missing conversion route from PCA to CA (Fig. 1). The PCA decarboxylase genes involved in the conversion of PCA to CA were searched. Since *K. pneumoniae-*derived PCA decarboxylases were successfully expressed in *E. coli* and *S. cerevisiae*^14,16^, two decarboxylase genes in *K. pneumoniae*, *kpdC* (*Klebsiella* *pneumoniae* decarboxylase encoding 4-hydroxybenzoate decarboxylase) and *aroY* (encoding PCA decarboxylase), were synthesized after codon-optimization for *C. glutamicum*. A modified plasmid named pMESK101 was constructed for the expression of target genes by the introduction of multiple cloning sites into pSK1Cat^33^. The *kpdC*^*opt*^ gene (KPN_03097p) and the *aroY*^*opt*^ gene (KPN_01161p) were cloned into pMSEK101 under the control of the *P*_180_ promoter, resulting in pMESK102 and pMESK103, respectively (Table 1, Fig. 2A). The *P*_180_ (180 bp intergenic fragment of cg2195 encoding putative membrane protein) was selected as a strong promoter and showed better homoserine acetyltransferase activity in *C. glutamicum*^33,34^. The *C. glutamicum* strains expressing pMESK102 and pMESK103 were named STR004 and STR005, respectively, and were tested to determine whether MA accumulated in their culture broths. However, MA accumulation did not occur in those strains (Fig. 2B, Supplementary Figs 2 and 4). It has been reported that the genes encoding decarboxylases, including PCA decarboxylase, require the co-expression of two other genes for full enzyme activity^35–37^. The co-expression of decarboxylase-associated proteins in a *P. putida* KT2440-based strain reduced PCA accumulation and increased the production of MA from aromatic lignin and/or glucose^17^. The decarboxylase found in *K. pneumoniae* is clustered with two further genes (*kpdB, kpdC, kpdD*) in the chromosome, *kpdB* and *kpdD* coding for the subunit and *kpdC* coding for 4-hydroxybenzoate decarboxylase. So three genes (*kpdB, kpdC, kpdD)* were codon-optimized in an original coupling form for plasmid-based overexpression and named pMESK104 (BCD^*opt*^). The *aroY*^*opt*^ gene was also synthesized for decarboxylase activity in the form of the *aroY, kpdB, kpdD* operon (YBD^opt^), and named pMESK105 as shown in Fig. 2A. The *aroE-pcaG/H-catB* triple knockout *Corynebacterium* strains containing pMESK104 and pMESK105, were generated as strains STR006 and STR007, respectively. The accumulation of MA, which was not seen before, was finally detected in both strains (Fig. 2B), although only a small concentration of MA was formed with still substantial concentrations of the intermediates DHS and PCA.

**Table 1:** Plasmids and strains used in this study.

Plasmid	Relevant characteristics	Ref.
pK19*mobsacB*	Kan^R^; vector for allelic exchange in *C. glutamicum*; (pK18 *oriVE.c*., *sacB*, *lacZ*α)	^56^
pK19aroE	pK19*mobsacB* carrying fragments of *aroE* gene upstream and downstream	This work
pK19pcaGH	pK19*mobsacB* carrying fragments of *pcaG* and *pcaH* gene upstream and downstream	This work
pK19catB	pK19*mobsacB* carrying fragments of *catB* gene upstream and downstream	This work
pMESK101	pSK1Cat modification vector including restriction sites (*Bam*HI*, BglII, Mun*I*, Nde*I*, Pst*I*, Sal*I*, Sca*I*, Spe*I*, and Xba*I), *oriT* and terminator	^57^, This work
pMESK102	pMESK101 carrying fragment of *P180*_ *kpdC*^*opt*^	This work
pMESK103	pMESK101 carrying fragment of *P180_ aroY*^*opt*^	This work
pMESK104	pMESK101 carrying fragment of *P*180_*kpdB kpdC kpdD*^*opt*^	This work
pMESK105	pMESK101 carrying fragment of *P*180_*aroY kpdB kpdD*^*opt*^	This work
pMESK106	pMESK101 carrying fragment of *Ptuf*_*kpdB kpdC kpdD*^*opt*^	This work
pMESK107	pMESK101 carrying fragment of *Ptuf*_*aroY kpdB kpdD*^*opt*^	This work
pMESK108	pMESK101 carrying fragment of *Psod*_*kpdB kpdC kpdD*^*opt*^	This work
pMESK109	pMESK101 carrying fragment of P*sod*_*aroY kpdB kpdD*^*opt*^	This work
**Strain**	**Relevant characteristics**	**Ref**.
*C. glutamicum* 13032	wild-type strain, biotin-auxotrophic	KCTC
STR001	*C. glutamicum* 13032 Δ*aroE*	This work
STR002	*C. glutamicum* STR001 Δ*pcaG*, Δ*pcaH*	This work
STR003	*C. glutamicum* STR002 Δ*catB*	This work
STR004	*C. glutamicum* STR003/pMESK102	This work
STR005	*C. glutamicum* STR003/pMESK103	This work
STR006	*C. glutamicum* STR003/pMESK104	This work
STR007	*C. glutamicum* STR003/pMESK105	This work
STR008	*C. glutamicum* STR003/pMESK106	This work
STR009	*C. glutamicum* STR003/pMESK107	This work
STR010	*C. glutamicum* STR003/pMESK108	This work
STR011	*C. glutamicum* STR003/pMESK109	This work

To increase the MA titer, promoter optimization for foreign decarboxylase expression was required to increase the efficiency of MA conversion. To select the strong promoter of the highly expressed genes, as well as the expression profiles of the genes involved in MA biosynthesis, transcriptome analysis was performed using the STR003 strain. Approximately 3100 genes were analyzed *via* RNA-sequencing and the genes with higher expression based on standardized transcription values (RPKM) were identified. The expression levels of the genes at 7 h (log-phase cells) and 12 h (stationary-phase cells) were sorted in order of highest expression in the STR003 strain (Supplementary Fig. 5). Based on the RNA-sequencing analysis, two strong promoters named *P*_*tuf*_ (promoter of cg0587 encoding elongation factor TU) and *P*_*sod*_ (promoter of cg3237 encoding superoxide dismutase) were selected, regardless of culture or growth conditions, which were then used for PCA decarboxylase gene expression. Plasmids were constructed to express two types of PCA decarboxylase/subunit gene under each of the selected promoters and named pMESK106 (*P*_*tuf*__BCD^opt^), pMESK107 (*P*_*tuf*__YBD^opt^), pMESK108 (*P*_*sod*__BCD^opt^) and pMESK109 (*P*_*sod*__YBD^opt^), respectively (Fig. 2A). Each strain (from STR008 to STR011), harboring each plasmid, was cultured in LB medium with glucose for 3 days. The production level of MA was different depending on the promoter, as shown in Fig. 2B. The highest MA titer was observed in strain STR011, which expressed *P*_*sod*__YBD^opt^. It yielded up to 340 mg/L of MA, a 150% increase compared to strain STR009 (*P*_*tuf*__YBD^opt^), which showed 220 mg/L of MA titer. DHS was accumulated in relatively large amount in all strains and only small amount of PCA was produced in strain STR008 and STR010, which were expressing BCD^opt^. In particular, when the complexes were compared under the same sod promoter, the PCA-to-MA conversion efficiency by the YBD^opt^ complex (STR011) was more effective than the BCD^opt^ complex (STR009).

### Medium optimization for MA production

**Figure 3:**
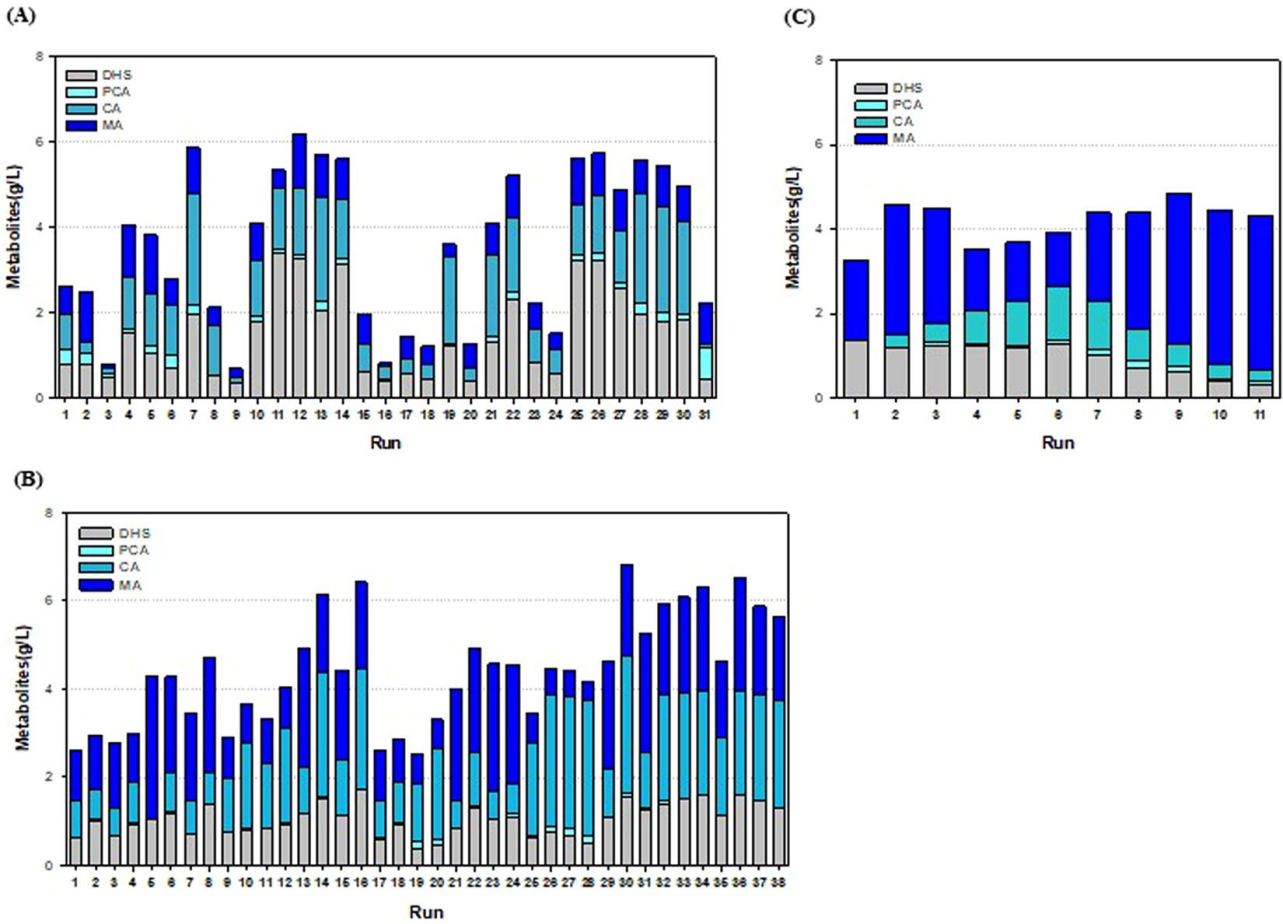
(**A**) Results of OFAT experiment with various nitrogen sources. Numbers represent each combination. (**B**) Results of the Full Factorial Design (FFD) experiment. (**C**) Results of the Steepest Ascent Method (SAM) experiment.

We choose a strain STR011 as a MA-producing *C. glutamicum* and this strain was used for medium optimization for large-scale fermentation. Medium optimization based on the CMP medium was investigated and glucose was used as a carbon and energy source. As glucose is suitable for sustainable production and low-cost biomass convertibility, it is beneficial for the microorganism-based mass production of chemicals^38^. Since the engineered strain used for MA production was deficient in *aroE*, provision of an optimized supply of aromatic amino acid in the medium was considered. Schulz *et al*. reported that in glutamate production using *C. glutamicum*, the regulation of nitrogen content resulted in a 5–7-fold difference between the activities of the two major enzymes^39^. Based on these outcomes, the nitrogen source, considered to have the most significant effect on MA production in the CMP medium, was preferentially investigated in this study. The “one factor at a time” (OFAT) method was used for optimization of the nitrogen source. This method was used to confirm the effects of one factor or variable at a time, while leaving the other factors unchanged^40^. Thus, the effects of various cell-culture medium components were quickly screened in the early stages of medium optimization. Thirty-one nitrogen sources were investigated in this study (Supplementary Table 3). The average production of total metabolites, according to the tested components, was approximately 3.54 g/L, and in the case of corn steep solid (CSS), the production was 6.17 g/L, which was 170% of the average value (Fig. 3A). The investigated medium comprised complex nutrients, i.e., casamino acid, yeast extract, and CSS, which enabled an increased production of the total metabolites. CSS, associated with the highest productivity, is a by-product obtained in the form of corn steep liquor (CSL) powder. CSS is rich in various vitamins and minerals and has been used as an industrial medium because it is inexpensive^41,42^.

The major effects of media compositions and their correlations must be logically analyzed to rapidly screen for the important factors influencing production. Efficiency in the design of medium optimization strategies helps to determine the factors affecting productivity and in modifying experiments, thus saving time and resources. The full factorial design (FFD) was used in this study for medium optimization. Unlike the OFAT method, the correlation levels of the factors can be confirmed *via* this method. Five factors (glucose, (NH_4_)_2_SO_4_, yeast extract, CSS, and KH_2_PO_4_), considered to be the most important for cell growth and productivity, were used for the test combinations (Supplementary Table 4). Analyses performed using a statistical program (Design-expert 6.0) revealed highly significant outcomes with P-values <0.001 (Supplementary Table 5). In combination #5, the level of total precursors was low, but the MA productivity was the highest (3.28 g/L) (Fig. 3B). The concentrations of glucose, (NH_4_)_2_SO_4_, CSS, and KH_2_PO_4_ were decreased, whereas the yeast extract concentration was increased, enhancing MA productivity. Among the factors analyzed, the effect of the yeast extract was the most significant, as confirmed *via* first-order polynomials suggested by the statistical program.

Based on the primary model obtained using ANOVA for the FFD design, medium optimization was conducted using the Steepest Ascent Method (SAM). SAM is a strategy of approaching the region of maximal production based on the results of FFD^40^. The new step values required for the SAM test were calculated using the polynomial parameter obtained from the FFD before the tests were conducted. Eleven steps were designed (Supplementary Table 6). In combinations #1 to #5, MA productivity gradually decreased and then gradually increased in combinations #6 and thereafter. The highest productivity was obtained in combination #9, at approximately 3.66 g/L of MA (Fig. 3C). In summary, MA production did not occur in combinations with a low concentration of yeast extract and a high concentration of CSS. However, production increased when a combination of a high concentration of yeast extract and a low concentration of CSS was used. Metabolite production patterns varied in accordance with the combinations of various concentrations of the medium components. Finally, the highest MA productivity was observed in the combination SL#9.

### Fed-batch fermentation in 7-L lab-scale and 50-L pilot-scale fermenter

**Figure 4:**
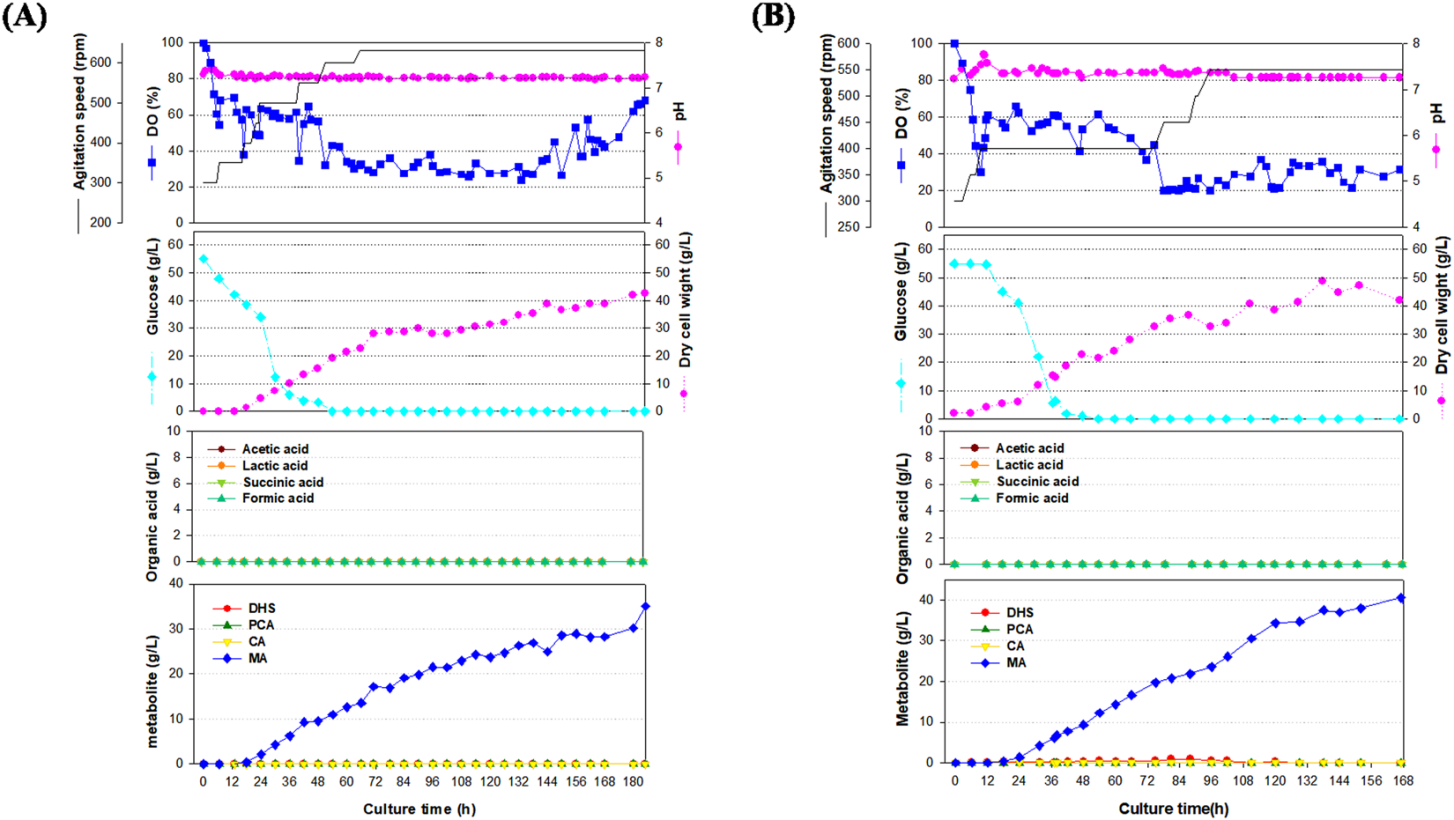
Time course profiles of cell growth, agitation speed, DO concentration, pH, DCW, glucose, organic acids and metabolite production by the STR011 strain in the 7-L fermenter. (**A**) The feeding medium was sequentially injected at cultivation time periods of 32, 44, 88, 95 and 162 h at a rate of 0.2268 mL/min, 0.3402 mL/min, 0.2268 mL/min, 0.2835 mL/min and 0.3402 mL/min. (**B**) The feeding medium was sequentially injected at cultivation time periods of 39, 52, 80, 85, 91.5, and 117 h at a rate of 0.2268 mL/min, 0.3402 mL/min, 0.2268 mL/min, 0.2835 mL/min, 0.3402 mL/min, 0.4536 mL/min. During the fed-batch cultivation, 230.2 g/L and 227 g/L of glucose was totally added, respectively.

**Figure 5:**
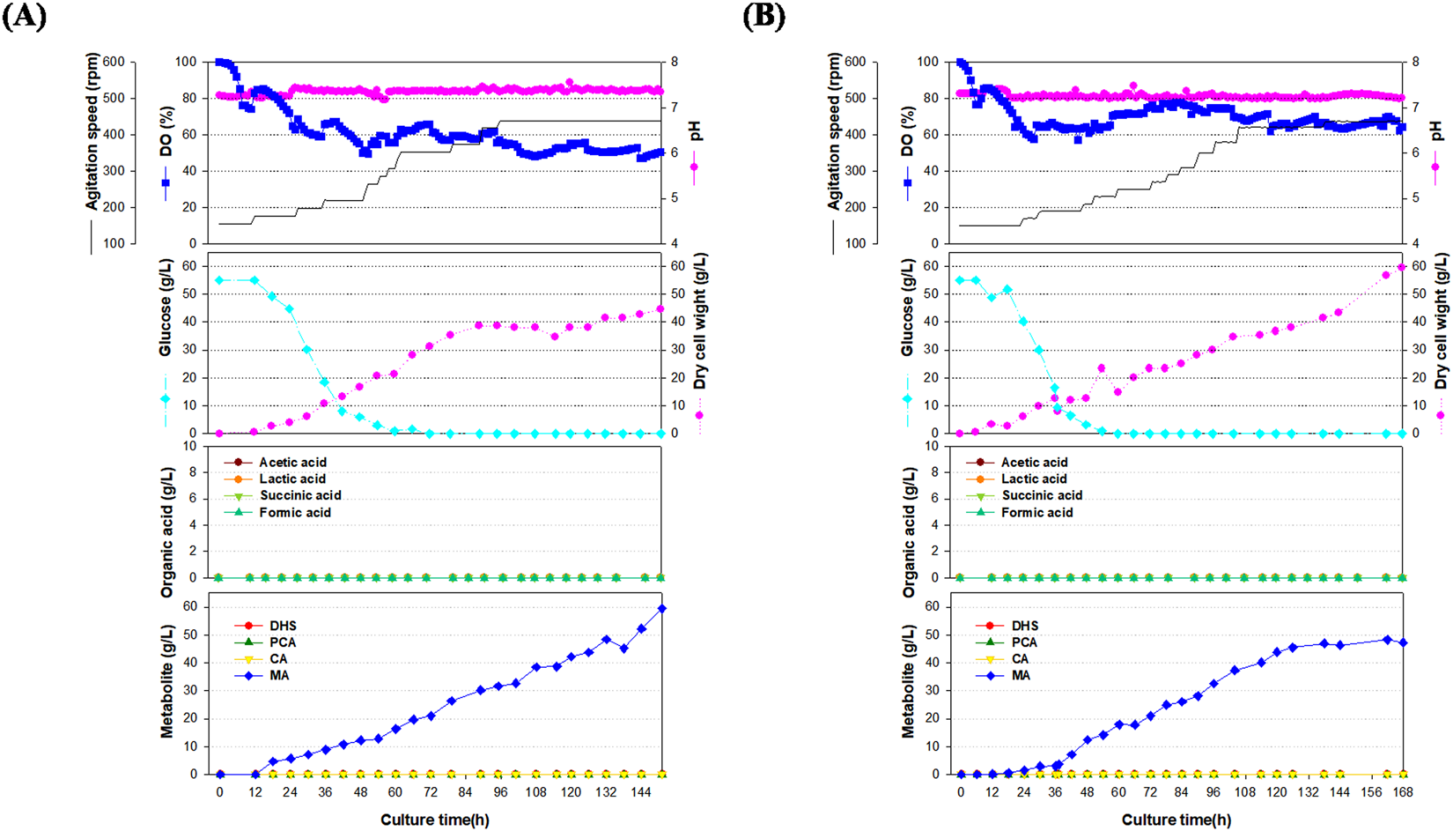
Time course profiles of cell growth, agitation speed, DO concentration, pH, DCW, glucose, organic acids and metabolite production by the STR011 strain in the 50-L fermenter. (**A**) The feeding medium was sequentially injected at cultivation time periods of 40, 60, 72, 95 and 116 h at a rate of 1.3608 mL/min, 2.0412 mL/min, 2.7216 mL/min, 3.0618 mL/min and 3.402 mL/min. (**B**) The feeding medium was sequentially injected at cultivation time periods of 37, 53, 76, 90, 104, 138, and 162 h at a rate of 1.3608 mL/min, 2.0034 mL/min, 2.7216 mL/min, 3.402 mL/min, 3.7422 mL/min, 4.2236 mL/min, 3.402 mL/min. During the fed-batch cultivation, 233.1 g/L and 259.8 g/L of glucose was totally added, respectively.

The fed-batch culture carried out using the STR011 strain, produced 3.66 g/L of MA in SL#9, which was obtained *via* media optimization. Thus, scaled-up operations, from shake flasks to fermenters, are largely influenced by the effects of operating conditions on gas-liquid mass transfer, shear stress, mixing time, antifoam, pH regulation, and power input^43^. To establish optimum conditions in the 7-L laboratory-scale fermenter, repetitive fermentation was optimized. The second growth culture medium was replaced with GF1, and the SL#9 production medium was modified with SLP medium (Supplementary Fig. 6). For the fed-batch culture, an SLP medium without phosphates was used as the feeding medium. After the glucose levels in the fermenter broth were estimated, feeding was repeated using a peristaltic pump to maintain levels above 5 g/L glucose. The addition of phosphates in the feeding medium facilitated cell growth, accompanied with decreased MA production. Thus, phosphates were eliminated from the feeding medium (Supplementary Fig. 7). The final composition of the feeding medium contained glucose (412.5 g/L), (NH_4_)_2_SO_4_ (46.095 g/L), yeast extract (103.8 g/L), MgSO_4_.7H_2_O (15 g/L), citric acid (8.5 g/L), and trace solution. (7.5 mL/L). Two independent 7-L laboratory-scale fed-batch fermentations were performed using final modified medium, and the results are shown in Fig. 4A and B. MA production steadily increased after 16 h of culturing, approaching final concentration of 35.0 g/L and 40.5 g/L, respectively. Metabolites such as DHS, PCA, and CA were negligible in the culture solution throughout the culture period. Furthermore, DCW showed a similar pattern of increase in MA production, eventually approaching a concentration of approximately 42 g/L. Interestingly, the trend in DCW increase was similar to that of MA production, indicating that MA production in the currently developed STR011 strain exhibited growth-associated production. Such a production pattern can be maximized when cultivation conditions are optimized based on production yield (*Y*_*p/s*_) and DCW yield (*Y*_*x/s*_). Cultivation processes such as fed-batch cultures are particularly likely to facilitate maximum production^44^. The actual values of *Y*_*p/s*_ and *Y*_*x/s*_ were similar (0.165 and 0.185 g DCW/g glucose, respectively), which implied the possibility of greatly improved production *via* cultural or metabolic engineering, with a maximized specific production yield (*Y*_*p/x*_) (Table 2). Moreover, the DO in the fermenter was maintained above 20% by controlling fermenter agitation to a maximum of 500~600 rpm. DO was shown to increase after 144 h of culturing, probably owing to an imbalance in broth composition to an excess of by-products. Based on the results of the 7-L fermenter, a pilot-scale fermentation process was carried out using a 50-L fermenter. Since *catA* requires oxygen to convert CA into MA and to produce high levels of DCW in the 7-L fermenter, adequate oxygen supply was determined to be a crucial factor in the fermentation process. In such scale-up processes, the volumetric mass transfer coefficient (*k*_*L*_*a*) was determined to be the most important scale-up factor during the evaluation of aeration efficiency in all fermenters^45–47^. For a successful scale-up, *k*_*L*_*a* was measured, following which six impeller blades and a sintered sparger were installed at the last step, which increased oxygen supply while decreasing shear stress. Consequently, the *k*_*L*_*a* values were approximately ranging from 0.01 to 0.06 s^−1^ in the 7-L and 50-L fermenters, when the agitation speed was increased from 100 to 350 rpm at an aeration rate of 1 vvm (Supplementary Fig. 8). In the 50-L fermenter (Fig. 5A and B), DCW continued to increase even after 12 h of culturing, eventually approaching 51.8 ± 7.2 g/L (average value of the duplicate). Moreover, MA production began with the initiation of cell growth, as seen in the 7-L fermenter, and two independent cultures exhibited final MA production of 59.4 g/L and 48.5 g/L, respectively (average value of the duplicate is 53.8 g/L). Since MA production continued to increase until the end of culturing, higher production levels can be anticipated through the development of a more enriched feeding medium. Higher MA production levels seen in the 50-L fermenter compared to the 7-L fermenter are probably the result of the sintered sparger, which maintained a DO value above 40% until the end of culturing, thereby increasing the oxygen supply to the cells, and improving MA production. According to the feeding strategy, increasing the agitation speed maintained a higher level of DO in the 50-L fermenter than in the 7-L fermenter throughout culturing. In other words, as the *k*_*L*_*a* value in the 50-L fermenter increased at 250 rpm or above, a higher level of DO was observed after 60 h of culturing, probably leading to greater MA production. The increased rate of oxygen supply also resulted in a 60% improvement (approximately) in specific MA production yield *(Y*_*p/x*_), a feature shared by cells that can produce MA, ultimately resulting in approximately 1.074 g MA/g DCW (Table 2). As in the case of the 7-L fermenter, glucose concentration was maintained at a level close to zero at an optimum feeding rate and no other metabolite (other than MA) was detected.

**Table 2:** **A**, 7-L lab-scale fermentation; **B**, 50-L pilot-scale fermentation; *P*_*f*_, maximum MA production; *X*_*f*_, maximum dry cell weight; *S*_*f*_, final residual glucose concentration; *Q*_*p*_, average volumetric MA production rate; *q*_*p*_, average specific MA production rate; *Y*_*p/x*_, specific MA production; *Y*_*p/s*_, MA production yield based on glucose; *Y*_*x/s*_, DCW yield based on glucose. The data represent the mean (**±**standard deviation) of two independent experiments.

	A (7-L)	B (50-L)
***P***_*f*_ (g IA/L)	37.7 ± 2.7	53.8 ± 5.5
***X***_*f*_ (g DCW/L)	45.3 ± 2.7	51.8 ± 7.2
***S***_*f*_ (g glucose/ L)	0	0
***Q***_*p*_ (g MA/L/hr)	0.216 ± 0.027	0.34 ± 0.053
***q***_*p*_ (g MA/g DCW/hr)	0.005 ± 0.001	0.006 ± 0.002
***Y***_*p/x*_ (g MA/g DCW)	0.892 ± 0.072	1.074 ± 0.256
***Y***_*p/s*_ (g MA/g glucose)	0.165 ± 0.013	0.197 ± 0.011
***Y***_*x/s*_ (g DCW/g glucose)	0.185 ± 0.001	0.192 ± 0.036

## Discussion

It was previously reported that the introduction of three heterologous biosynthetic genes such as *aroZ, aroY*, and *catA* into the *aroE*-deleted *E. coli*, led to the accumulation of MA as a final product, through the DHS-PCA-CA-MA route. However, the same strategy cannot be applied to *C. glutamicum*, since the orthologs of *aroZ* and *catA* are present in the chromosome. Moreover, both PCA and MA could be further metabolized through bypass routes. The β-ketoadipate pathway present in *C. glutamicum* is able to decompose PCA into acetyl-CoA or succinyl-CoA^48^. Moreover, the presence of *catB* (chloromuconate cycloisomerase) in *C. glutamicum* is able to metabolize MA to muconolactone. In this work, construction of an MA-producing *C. glutamicum* cell factory was designed by the introduction of PCA decarboxylase together with its subunit genes, as well as applying a chromosome engineering strategy through the disruption of *aroE, pcaG/H*, and *catB*, to accumulate the precursors necessary for MA biosynthesis.

Sonoki *et al*. described PCA decarboxylation as a rate-limiting step in MA production and found that plasmid-based co-expression of *kpdB* and/or *kpdD* increased the activity of PCA decarboxylase, eliminating the bottleneck, as compared to AroY expression alone in *E. coli*^49^. Johnson *et al*. successfully enhanced MA production in a *P. putida* TK2440-derived strain *via* the co-expression of decarboxylase-associated proteins, reducing PCA accumulation relative to strains expressing PCA decarboxylase alone^17^. Although the function of B and D proteins was previously unknown, it was recently discovered that homologues of B protein synthesize a cofactor required for the activity of decarboxylases^50–52^. In our study of *C. glutamicum*, the conversion from PCA to CA by PCA decarboxylase was observed only in the presence of its two subunit genes, co-expressed in the operon system. The MA production level of the codon-optimized PCA decarboxylase under the *P*_*sod*_ promoter was higher than for those under the *P*_*tuf*_ and *P*_180_ promoters, even though RNA sequencing results showed that cg0587 (*tuf)* showed the highest transcription. RBS engineering of the *sod* promoter system was also conducted based on the results confirming an enhanced gene activity through RBS engineering for the genes in the aromatic amino acid-biosynthetic pathway, which confirmed the increase in shikimate production^26^. The native RBS in the *sod* promoter system was engineered to test 12 combinations, as described in their report, although this did not result in increased MA production (Supplementary Fig. 9). This may imply that the native RBS of the *sod* promoter was already optimal for the expression of the YBD^opt^ complex. In addition, the production efficiency of intermediates including MA was better when expressed in the form of the YBD^opt^ complex than in the translationally coupled BCD^opt^ complex.

Glucose was selected as the carbon and energy source for MA production to improve the cost-effectiveness and industrial production process, and 31 types of nitrogen sources were screened with the OFAT method to determine the optimal nitrogen source. Most nitrogen sources that yielded high metabolite production and MA were complex compounds such as casamino acid, corn steep solids, and yeast extracts (Fig. 3A). This appears to be associated with the characteristics of amino acid auxotrophs, which were deficient in *aroE*. In addition, the presence of sufficient amino acids, provided by the complex ingredients, accelerated cell growth, and subsequent metabolite production presumably increased. Furthermore, metabolite production rates varied as much as six times among the substances, depending on the nitrogen source in the culture medium. We therefore confirmed that the nitrogen source is an important factor in the production of MA.

To evaluate the interaction between two factors, we employed a fractional factorial design (FFD) and obtained significant results. These were expressed as the following mass balance: MA = +1.65 − 0.12*A − 0.033*B + 0.75*C − 0.16*D − 0.049*E + 0.058*A*B − 0.058*A*C − 0.026*A*D + 2.170E - 003*A*E + 8.868E - 003*B*C + 0.015*B*D + 0.032*B*E − 0.017*C*D + 0.14*C*E + 0.016*D*E (A, glucose; B, (NH_4_)_2_SO_4_, C, yeast extract; D, corn steep solid; E, KH_2_PO_4_). The five factors selected, in addition to the yeast extract and CSS, which were suggested in the OFAT experiment, were included in the FFD design. In addition, the concentration range of each ingredient was determined based on the results of additional experiments (Supplementary Fig. 8). Yeast extract with a *coefficient* of 0.75 had the greatest effect on the production rate and a positive value, which implied an increase in concentration, whereas CSS had a *coefficient* of 0.16 and a negative value, implying a decline in concentration. Thus, as each culture medium component comprising a carbon source, a nitrogen source, and various other substances did not play an independent role but interacted with other components, it was critical to identify their optimal concentrations. The response surface method (RSM), based on the quadratic model of a central composite design (CCD), allows for the evaluation of interactions among all components. Thus, it can statistically lead to maximal production. CCD requires multiple experiments to obtain statistically significant results. Generally, the number of experiments in an experimental design for culture medium optimization is determined using the equation 2^*k*^ + 2*k* + central point (*k* = individual factor, central point >4)^40^. Therefore, considering the accuracy and reproducibility requirement of the experiments, it would be difficult to evaluate more than four factors at a time. In contrast, the SAM design is a statistical experimental method that efficiently evaluates the optimal culture medium concentration based on the FFD design. The center point used in the FFD design showed a DHS production rate of 1 g/L and an MA production rate of 2 g/L. The SAM experiment resulted in an MA production rate of 3.66 g/L, a 10-fold increase, essentially inhibiting DHS production (Fig. 3C). Therefore, the process of culture medium optimization enabled decreasing the production of intermediate metabolites and identifying the optimal culture medium concentration that maximized MA production. In addition, a metabolic engineering approach involving the further investigation of genes associated with increased production and final culture medium optimization process using RSM is expected to contribute to further enhancements in MA production.

To develop a large-scale fermentation process for MA-producing strains, we carried out a batch fermentation, which produced 10 g/L of MA (Supplementary Fig. 6). The engineered strains required a supply of amino acids in the culture medium because they were deficient in *aroE*, and for optimal regulation of a balance between cell growth and MA production, through optimal culture medium conditions. Fed-batch culture is a standard method that produces various metabolites, as it promotes high cell density, strictly regulates the specific growth rate (*μ*) and substrate concentration, and suppresses the production of organic acids by effectively controlling DO levels. A culture producing 35 g/L of MA was developed *via* modification of the second growth culture and regulation of the feeding medium in 7-L laboratory-scale fed-batch cultivation. In case of metabolically engineered *E. coli*, the Drath *et al*. reported the carbon yield of 22% (mol/mol) in M9 defined medium. On the contrary, our *C. glutamicum* cells were observed to produce no MA in the M9 medium until the end of fermentation, almost no cell growth as well. In the statistically optimized complex medium, the *C. glutamicum* strain showed a slightly lower carbon yield of 19.5% (mol/mol), thus revealing significantly different fermentation physiology compared to the *E. coli* strain developed in the Drath laboratory^2^. In the aromatic amino acids process, *E. coli* and *C. glutamicum* have characteristic secondary metabolites that suppress cell density at certain concentrations^53,54^. In contrast, an MA-producing culture has characteristic primary metabolites because of which, increased cell growth is directly correlated with MA production, probably due to the absence of *aroE*, and cell growth is determined by optimal levels of aromatic amino acids in the culture medium. Although MA production is directly related to cell growth, the high cell density in a fermenter interferes with mass transfer; therefore, an optimized and accurate feeding strategy may be required. We observed that cell growth and primary metabolite fermentation persisted in the 50-L fermenter and produced 53.8 ± 5.6 g/L MA after culturing. In contrast to the 7-L fermenter, agitation consistently increased after the initial 60 h of culturing in the 50-L fermenter, and its *k*_*L*_a was higher than that of the 7-L fermenter (Supplementary Fig. 8). *k*_*L*_a values were not measured at higher agitation levels in the two fermenters. However, considering that a 2-fold increase in DO was confirmed in the 50-L fermenter compared with that in the 7-L fermenter, the increased rates of oxygen transfer could positively affect MA production. Numerous studies have already confirmed the effects of high oxygen concentrations in aerobic fermentation-based production cultures. Strain development *via* metabolic engineering, medium optimization, and the development of accurate fed-batch cultivation, are expected to facilitate the establishment of an industrial production process using *C. glutamicum*.

In summary, we have constructed *C. glutamicum* strains capable of producing MA at high concentrations from D-glucose. To accumulate high concentrations of DHS and PCA, which are essential precursors for MA biosynthesis, shikimate/quinate dehydrogenase (*aroE*) and PCA deoxygenase subunits (*pcaG/H*) genes were deleted. The *catB* (chloromuconate cycloisomerase) gene was additionally deleted to prevent degradation of MA, but no accumulation of MA was observed in the *C. glutamicum* STR003 strain, which produced a small amount of MA only under specific conditions. MA was finally produced by heterologous expression of the PCA decarboxylase gene and subunit genes from *K. pneumoniae*, to complete the conventional pathway for MA biosynthesis *via* DHA, PCA, and CA. The STR011 strain expressing the *aroY*^*opt*^ gene with subunit genes under the *sod* promoter, showed the highest MA production. After several steps of medium optimization, 7-L and 50-L fed-batch fermentation was performed using optimized medium. It is the first time that MA has been produced in 50-L fed-fermentation in an engineered *C. glutamicum* strain, and the highest level of MA production yet demonstrated, at 59.4 g/L. A variety of engineering strategies to increase intracellular levels of PEP (phosphoenolpyruvate) and E4P (erythrose 4-phosphate), key intermediates of the shikimate pathway, and additional engineering to efficiently utilize various carbon sources including glucose, will lead us to obtain more efficient MA-producing *Corynebacterium* cell factories. Recently further engineered strain with deletion of the *iolR* gene which greatly increased glucose uptake has been developed, which had non-phosphotransferase system with increased PEP availability, showing improved MA production yield in the flask culture^55^.

## Methods

### Bacterial strains, media, and cultivation conditions

The bacterial strains and plasmids used in this study are listed in Table 1. For genetic manipulations, *E. coli* strains were grown at 37 °C in Luria-Bertani (LB) medium. *C glutamicum* ATCC13032 and mutants were cultured at 30 °C in LB with 8 g/L of glucose and Brain Heart Infusion (BHI). When needed, kanamycin, at a final concentration of 50 μg/mL, was used in the medium for the cultivation of *C. glutamicum* or *E. coli*. For preparation of *Corynebacterium* competent cells, BHI broth containing 91 g/L sorbitol was used^56^.

For MA production in flasks, the *C. glutamicum* strain was inoculated into LBG medium (20 g/L glucose in LB) and cultured at 30 °C for 16 h. One percent (v/v) of the inoculum was transferred into the second growth culture for 6 h under the same conditions. Next, the seed was transferred into a 250-mL flask with a 30-mL working volume (1% v/v inoculums). Production cultivation in flasks was performed for approximately 3–4 days at 30 °C with mixing at 240 rpm. Small-scale culture was also carried out using the same method as for flask cultivation, and cultured to a final volume of 1.3 mL in a 24-well plate in an 80% humidity chamber at 30 °C. For the production of metabolites and/or MA, CMP medium prepared in our laboratory was used prior to medium optimization. The CMP medium included: 60 g/L glucose, 10 g/L (NH_4_)_2_SO_4_, 5 g/L yeast extract, 10 g/L KH_2_PO_4_, 2 g/L MgSO_4_∙7H_2_O, 1.14 g/L citric acid, 1.68 g/L NaH_2_PO_4_, 5.112 g/L Na_2_HPO_4_, 1 mL/L trace metals, and 200 μg/L thiamine hydrochloride. The trace metal solution contained 2 mg/L FeSO_4_∙7H_2_0, 5 mg/L ZnSO_4_∙7H_2_0, 1 mg/L MnC1_2_∙4H_2_0, 0.55 mg/L Na_2_MoO_4_, and 4 mg/L CuSO_4_∙5H_2_0 in 1 L water, adjusted to pH 7.2 with 5 N NaOH. Media optimization was conducted using CMP medium as a starting point.

### Construction of plasmids and strains

General DNA manipulations were performed according to the method previously described^56^. The plasmids used in this study are listed in Table 1 and the primers are listed in Supplementary Table 1. Plasmid pMESK101 was modified from pSK1Cat^33^ to carry a multiple cloning site, *rrnB* T1 terminator, and *oriT*. To delete the *Xba*I restriction enzyme site in the pSK1Cat plasmid, the *kan*^*R*^ gene and pMB1 origin of replication were amplified from pSK1Cat, using the primer pair pMESK101_F and pMESK101_R, introducing *Spe*I and *Bgl*II restriction sites. The PCR products of the *kan*^*R*^ gene and the pMB1 origin of replication site were digested by *Spe*I and *Bgl*II, and pSK1Cat was digested by *Xba*I and *Bgl*II. The digested fragments were then ligated together. The multiple cloning site, terminator, and *oriT* were synthesized to introduce the deleted *Xba*I restriction enzyme site into pSK1Cat. The fragment, including nine kinds of restriction enzyme sites (*Bam*HI, *Bgl*II, *Mun*I, *Nde*I, *Pst*I, *Sal*I, *Sca*I, *Spe*I, and *Xba*I), *rrnB* T1 terminator, and *oriT*, was synthesized with *Bgl*II and *Kpn*I restriction site ends. The pSKCat1-deleted *Xba*I restriction enzyme site and the fragment were digested by *Bgl*II and *Kpn*I and ligated, generating pMESK101. *YBD*^*opt*^-carrying fragments of *aroY*^*opt*^ with *Nde*I and *Bam*HI ends, and *kpdB*^*opt*^ and *kpdD*^*opt*^ with *Bam*HI and *Xba*I ends, were synthesized as codon-optimized gene versions from Cosmo Genetech, Korea. BCD^*opt*^-carrying fragments of *kpdB*^*opt*^, *kpdC*^*opt*^ and *kpdD*^*opt*^ with *Bam*HI and *Xba*I ends, were also synthesized as codon-optimized gene versions from Cosmo Genetech, Korea. The fragment of YBD^*opt*^ and BCD^*opt*^ were ligated to pMESK101. Promoters were amplified from the genome of *C. glutamicum* ATCC 13032, using the primer pairs listed in Supplementary Table 1. The amplified promoters including *Spe*I and *Nde*I sites were ligated to the site of *Spe*I and *Nde*I of pMESK101, generating pMESK106, pMESK107, pMESK108 and pMESK109, respectively.

### Gene deletion

Chromosomal gene deletion was achieved *via* a markerless system using the suicide vector pK19mobsacB carrying the *sacB* gene^57^. Plasmids were transformed into *C. glutamicum* by electroporation and conjugation. The plasmid pK19aroE was constructed by amplifying the upstream and downstream DNA fragments of *aroE* of *C. glutamicum*, using primer sets of *aroE*_up_F/*aroE*_up_R and *aroE*_down_F/*aroE*_down_R, respectively, and inserting them into the pK19mobsacB vector, respectively. All PCR products were digested by the corresponding restriction enzymes and ligated with the pK19*mobsacB* vector digested by *Pst*I, *Sal*I, and *Eco*RI, generating the pK19aroE plasmid. pK19aroE was introduced via conjugation of *E. coli* S17-1 with *C. glutamicum* ATCC 13032. The single recombinants of pK19aroE in the chromosome of *C. glutamicum* strains were selected on BHI agar containing 50 mg/L kanamycin, and confirmed by PCR using primer pairs of *aroE*_con_F and *aroE*_con_R. These single colonies were inoculated into 50 mL LB broth without antibiotic and incubated at 30 °C for 12 h to allow excision. Appropriate dilutions were spread onto LB agar plates containing 10% sucrose, and single colonies were obtained and streaked onto BHIA agar plates containing 10% sucrose without antibiotics or kanamycin and grown at 30 °C for 24 h. The cells grown on the plates without antibiotics, but not on the plates with kanamycin, were chosen, and selected colonies confirmed by PCR were designated as the *C. glutamicum* STR001 strain. The *pcaG*/*H* and *catB* genes were deleted from the genome of *C. glutamicum* by the same method, resulting in strains of *C. glutamicum* STR002 and *C. glutamicum* STR003, respectively.

### RNA sequencing

For RNA sequencing, the *C. glutamicum* STR003 strain was grown in 50 mL LB media containing 8 g/L of glucose for seed culture, and in 500 mL LB media containing 8 g/L of glucose for the main culture. The same strain was cultured in LB medium without glucose at the same time as a control. Growth curves were obtained by culturing the strain at each condition, and strains at the log-phase and stationary-phase were sampled, respectively. Cells were collected at 7 h (log phase) and 12 h (stationary phase), and stored at −80 °C. The frozen cells were sheared in a mortar, and an RNeasy mini prep kit was used for RNA isolation, according to the manufacturer’s instructions. DNaseI treatment was used to eliminate possible chromosomal DNA contamination. RNA-Seq was performed by ChunLab Inc., Korea.

RNA-seq data have been deposited in the ArrayExpress database at EMBL-EBI (www.ebi.ac.uk/arrayexpress) under the accession number E-MTAB-7350.

### Metabolite analysis

*C. glutamicum* cells were removed from cultures by centrifugation and culture broth was purified using a membrane filter. The metabolites were separated by HPLC (Shimazu) using an Aminex HPX-87H column (Bio-Rad). The column was eluted with 5 mM H_2_SO_4_ as the mobile phase, at a flow rate of 0.6 mL/min and a temperature of 65 °C. MA, PCA, and DHS were analyzed at wavelengths of 262 nm, 259 nm, and 236 nm, respectively. Organic acids, including succinic acid, acetic acid, formic acid, and lactic acid, were detected at wavelengths of 210 nm.

### 7-L and 50-L fed-batch fermentation

The medium used in the 7-L culture was modified from that used for the flask cultures. The 1^st^ culture grown in GF1 medium was inoculated into the 2^nd^ growth culture, and the composition of GF1 medium was as follows: 30 g/L glucose, 6.5 g/L (NH_4_)_2_SO_4_, 3.75 g/L yeast extract, 5.5 g/L corn steep solid, 6.25 g/L KH_2_PO_4_, 2 g/L MgSO_4_∙7H_2_O, 1.14 g/L citric acid, 1 ml/L trace metals, and 200 µg/L thiamine hydrochloride. The second culture was inoculated into a 7-L fermenter (10% v/v inoculums) and/or 50-L fermenter (10% v/v inoculums) for the production culture. Production culture was conducted based on the SLP medium modified from SL#9 using 7-L (working volume: 3-L) and 50-L (working volume: 18-L). The SLP medium includes: 55 g/L glucose, 4.39 g/L (NH_4_)_2_SO_4_, 13.84 g/L yeast extract, 4.26 g/L KH_2_PO_4_, 2 g/L MgSO_4_∙7H_2_O, 1.14 g/L citric acid, 1 ml/L trace metals, and 200 µg/L thiamine hydrochloride. The trace metal solution was identical to that used in the CMP medium. The pH levels were maintained at 7.3 in each culture (10 N NaOH, 3 M HCl), and the DO level was maintained at 50 to 60% by controlling the agitation, aeration, and feeding rates. The configuration of each fermenter was as follows: 7-L fermenter; 2 impellers with 6 blades, ring typed sparger of 12 holes, bottom-driven, 160 mm of tank diameter, and 50-L fermenter; 3 impellers with 8 blades, ring typed sparger of 12 holes, top-driven, 310 mm of tank diameter. For determining volumetric oxygen mass transfer coefficient (*k*_*L*_a) in the 7-L and 50-L fermenter systems, a gas analyzer (Autolab-LK930A from Lokas, South Korea) was connected^58^. Fed-batch cultivation was conducted based on the initial feeding medium that was 7.5 times more concentrated than the SLP medium and phosphate was not added for the regulation of cell growth. The feeding medium was supplied when glucose was depleted, using a peristaltic pump.

## Electronic supplementary material

supplementary data

## References

[1] Curran KA, Leavitt JM, Karim AS, Alper HS (2013). Metabolic engineering of muconic acid production in *Saccharomyces cerevisiae*. Metab. Eng..

[2] Xie N, Liang H, Huang R, Xu P (2014). Biotechnological production of muconic acid: current status and future prospects. Biotechnol. Adv..

[3] Sato K, Aoki M, Noyori RA (1998). “Green” Route to Adipic Acid: Direct Oxidation of Cyclohexenes with 30 Percent Hydrogen Peroxide. Science.

[4] Jan CJB, Cavallaro S (2015). Transiting from Adipic Acid to Bioadipic Acid. 1, Petroleum-Based Processes. Ind Eng Chem Res.

[5] McKague AB (1999). Synthesis of Muconic Acids by Peracetic Acid Oxidation of Catechols. Synth. Commun..

[6] Neidle EL, Hartnett C, Bonitz S, Ornston LN (1988). DNA sequence of the *Acinetobacter calcoaceticus* catechol 1,2-dioxygenase I structural gene *catA*: evidence for evolutionary divergence of intradiol dioxygenases by acquisition of DNA sequence repetitions. J. Bacteriol..

[7] Warhurst AM, Clarke KF, Hill RA, Holt RA, Fewson CA (1994). Production of catechols and muconic acids from various aromatics by the styrene-degrader *Rhodococcus rhodochrous* NCIMB 13259. Biotechnol. Lett..

[8] Wu XC, Wang ZY, Zhou J, Zhu XF, Qian KX (2004). Studies of increasing the forward-mutation rate of UV irradiated *Streptomyces* sp. AP 19-1, an antibiotic producing strain. Yi Chuan.

[9] Seo J, Keum Y, Li QX (2009). Bacterial Degradation of Aromatic Compounds. Int. J. Environ. Res. Public Health.

[10] Vogt C, Kleinsteuber S, Richnow HH (2011). Anaerobic benzene degradation by bacteria. Microb. Biotechnol..

[11] Jung HM, Jung MY, Oh MK (2015). Metabolic engineering of *Klebsiella pneumoniae* for the production of *cis,cis*-muconic acid. Appl. Microbiol. Biotechnol..

[12] Wang J, Zheng P (2015). Muconic acid production from glucose using enterobactin precursors in *Escherichia coli*. J. Ind. Microbiol. Biotechnol..

[13] Okamura-Abe Y (2016). Beta-ketoadipic acid and muconolactone production from a lignin-related aromatic compound through the protocatechuate 3, 4-metabolic pathway. J. Biosci. Bioeng..

[14] Draths KM, Frost JW (1994). Environmentally compatible synthesis of adipic acid from D-glucose. J. Am. Chem. Soc..

[15] Niu W, Draths KM, Frost JW (2002). Benzene-free synthesis of adipic acid. Biotechnol. Prog..

[16] Weber C (2012). Biosynthesis of *cis,cis*-Muconic Acid and Its Aromatic Precursors, Catechol and Protocatechuic Acid, from Renewable Feedstocks by *Saccharomyces cerevisiae*. Appl. Environ. Microbiol..

[17] Johnson CW (2016). Enhancing muconic acid production from glucose and lignin-derived aromatic compounds *via* increased protocatechuate decarboxylase activity. Metab. Eng. Comm..

[18] Thompson B, Pugh S, Machas M, Nielsen DR (2018). Muconic Acid Production *via* Alternative Pathways and a Synthetic “Metabolic Funnel”. ACS Synth. Biol..

[19] Barton N (2018). Enabling the valorization of guaiacol-based lignin: Integrated chemical and biochemical production of *cis,cis*-muconic acid using metabolically engineered *Amycolatopsis* sp ATCC 39116. Metab. Eng..

[20] Sun X, Lin Y, Huang Q, Yuan Q, Yan Y (2013). A Novel Muconic Acid Biosynthesis Approach by Shunting Tryptophan Biosynthesis *via* Anthranilate. Appl. Environ. Microbiol..

[21] Lin Y, Sun X, Yuan Q, Yan Y (2014). Extending shikimate pathway for the production of muconic acid and its precursor salicylic acid in *Escherichia coli*. Metab. Eng..

[22] Sengupta S, Jonnalagadda S, Goonewardena L, Juturu V (2015). Metabolic engineering of a novel muconic acid biosynthesis pathway *via* 4-hydroxybenzoic acid in *Escherichia coli*. Appl. Environ. Microbiol..

[23] Dhar KS, Wendisch VF, Nampoothiri KM (2016). Engineering of *Corynebacterium glutamicum* for xylitol production from lignocellulosic pentose sugars. J. Biotechnol..

[24] Lee, J., Saddler, J. N., Um, Y. & Woo, H. M. Adaptive evolution and metabolic engineering of a cellobiose- and xylose- negative *Corynebacterium glutamicum* that co-utilizes cellobiose and xylose. *Microb. Cell. Fact*. **15**, 20-016-0420-z (2016). 10.1186/s12934-016-0420-z PMC4722713 26801253

[25] Chen Z (2017). Metabolic engineering of *Corynebacterium glutamicum* for the production of 3-hydroxypropionic acid from glucose and xylose. Metab. Eng..

[26] Zhang B (2015). Ribosome binding site libraries and pathway modules for shikimic acid synthesis with *Corynebacterium glutamicum*. Microb. Cell. Fact..

[27] Zhang, B., Liu, Z. Q., Liu, C. & Zheng, Y. G. Application of CRISPR*i* In *Corynebacterium glutamicum* for shikimic acid production. *Biotechnol. Lett*. **38**, 2153–2161 (2016). 10.1007/s10529-016-2207-z 27623797

[28] Kogure T, Kubota T, Suda M, Hiraga K, Inui M (2016). Metabolic engineering of *Corynebacterium glutamicum* for shikimate overproduction by growth-arrested cell reaction. Metab. Eng..

[29] Yoshikawa N, Mizuno S, Ohta K, Suzuki M (1990). Microbial production of *cis,cis*-muconic acid. J. Biotechnol..

[30] Liu WH, Li RM, Kung KH, Cheng TL (2003). Bioconversion of benzoic acid to *cis, cis*-muconic acid by *Corynebacterium pseudodiphtheriticum*. Food. Sci. Agric. Chem..

[31] Kubota T, Tanaka Y, Hiraga K, Inui M, Yukawa H (2013). Characterization of shikimate dehydrogenase homologues of *Corynebacterium glutamicum*. Appl. Microbiol. Biotechnol..

[32] Brzostowicz PC, Reams AB, Clark TJ, Neidle EL (2003). Transcriptional Cross-Regulation of the Catechol and Protocatechuate Branches of the ß-Ketoadipate Pathway Contributes to Carbon Source-Dependent Expression of the *Acinetobacter* sp. Strain ADP1 *pobA* Gene. Appl. Environ. Microbiol..

[33] Park SD (2004). Isolation and characterization of transcriptional elements from *Corynebacterium glutamicum*. J. Microbiol. Biotechnol..

[34] Park SD (2005). Derepression of a Methionine Biosynthetic Gene by Utilizing a Promoter Isolated from *Corynebacterium glutamicum*. Kor. J. Microbiol..

[35] Matsui T, Yoshida T, Hayashi T, Nagasawa T (2006). Purification, characterization, and gene cloning of 4-hydroxybenzoate decarboxylase of *Enterobacter cloacae* P240. Arch. Microbiol..

[36] Lupa B, Lyon D, Gibbs MD, Reeves RA, Wiegel J (2005). Distribution of genes encoding the microbial non-oxidative reversible hydroxyarylic acid decarboxylases/phenol carboxylases. Genomics.

[37] Lupa B, Lyon D, Shaw LN, Sieprawska-Lupa M, Wiegel J (2008). Properties of the reversible nonoxidative vanillate/4-hydroxybenzoate decarboxylase from *Bacillus subtilis*. Can. J. Microbiol..

[38] Binder JB, Raines RT (2010). Fermentable sugars by chemical hydrolysis of biomass. Proc. Natl. Acad. Sci. USA.

[39] Schulz AA, Collett HJ, Reid SJ (2001). Nitrogen and carbon regulation of glutamine synthetase and glutamate synthase in *Corynebacterium glutamicum* ATCC 13032. FEMS Microbiol. Lett..

[40] Singh, V. *et al*. Strategies for Fermentation Medium Optimization: An In-Depth Review. *Front. Microbiol*. **7**, 10.3389/fmicb.2016.02087 (2016). 10.3389/fmicb.2016.02087 PMC5216682 28111566

[41] Vandamme, E. J. In *Biotechnology for Agro-Industrial Residues Utilisation: Utilisation of* Agro-Residues (eds Nigam, S. Poonam & Pandey, A.) 3–11 (Springer Netherlands, Dordrecht, 2009).

[42] Posten, C. H. & Cooney, C. L. In *Growth of Microorganisms (with Diagram)* 111–162 (Wiley-VCH Verlag GmbH, 1993).

[43] Seletzky JM (2007). Scale-up from shake flasks to fermenters in batch and continuous mode with *Corynebacterium glutamicum* on lactic acid based on oxygen transfer and pH. Biotechnol. Bioeng..

[44] Kamp AV, Klamt S (2017). Growth-coupled overproduction is feasible for almost all metabolites in five major production organisms. Nat. Commun..

[45] Margaritis A, Zajic JE (1978). Mixing, mass transfer, and scale-up of polysaccharide fermentations. Biotechnol. Bioeng..

[46] Chun G, Agathos S (2001). Application of Oxygen Uptake Rate Measured by a Dynamic Method for Analysis of Related Fermentation Parameters in Cyclosporin A Fermentation: Suspended and Immobilized Cell Cultures. J. Microbiol. Biotechnol..

[47] Shukla VB, Parasu Veera U, Kulkarni PR, Pandit AB (2001). Scale-up of biotransformation process in stirred tank reactor using dual impeller bioreactor. Biochem. Eng. J..

[48] Brinkrolf K, Brune I, Tauch A (2006). Transcriptional regulation of catabolic pathways for aromatic compounds in *Corynebacterium glutamicum*. Genet. Mol. Res..

[49] Sonoki, T. *et al*. Enhancement of protocatechuate decarboxylase activity for the effective production of muconate from lignin-related aromatic compounds. *J. Biotechnol*. **192** Pt A, 71–77 (2014). 10.1016/j.jbiotec.2014.10.027 25449108

[50] Lin F, Ferguson KL, Boyer DR, Lin XN, Marsh EN (2015). Isofunctional enzymes PAD1 and UbiX catalyze formation of a novel cofactor required by ferulic acid decarboxylase and 4-hydroxy-3-polyprenylbenzoic acid decarboxylase. ACS Chem. Biol..

[51] Payne KA (2015). New cofactor supports alpha,beta-unsaturated acid decarboxylation via 1,3-dipolar cycloaddition. Nature.

[52] White MD (2015). UbiX is a flavin prenyltransferase required for bacterial ubiquinone biosynthesis. Nature.

[53] Rodriguez A (2014). Engineering *Escherichia coli* to overproduce aromatic amino acids and derived compounds. Microb Cell Fact..

[54] Cheng L (2012). Effect of feeding strategy on l-tryptophan production by recombinant *Escherichia coli*. Ann. Microbiol..

[55] Shin W (2018). Characterization of a non-phosphotransferase system for *cis,cis*-muconic acid production in *Corynebacterium glutamicum*. Biochem. Biophys. Res. Commun..

[56] Sambrook, J. F. & Maniatis, T. In *Molecular Cloning: A Laboratory Manual* (Cold Spring Harbor Laboratory Press, Cold Spring Harbor, NY., 1989).

[57] Schäfer A (1994). Small mobilizable multi-purpose cloning vectors derived from the *Escherichia coli* plasmids pK18 and pK19: selection of defined deletions in the chromosome of *Corynebacterium glutamicum*. Gene.

[58] Shin WS, Lee D, Kim S, Jeong YS, Chun GT (2013). Application of scale-up criterion of constant oxygen mass transfer coefficient (kLa) for production of itaconic acid in a 50 L pilot-scale fermentor by fungal cells of *Aspergillus terreus*. J. Microbiol. Biotechnol..

