# Supplementary material for: Corynebacterium Cell Factory Design and Culture Process Optimization for Muconic Acid Biosynthesis
Source: Sci Rep. 2018 Dec 21;8:18041. doi: 10.1038/s41598-018-36320-4 (PMC6303301; doi:10.1038/s41598-018-36320-4)
Supplement: Supplementary file 1 — supplementary data [file 41598_2018_36320_MOESM1_ESM.docx]

**Supplementary Information**

***Corynebacterium* Cell Factory Design and Culture Process Optimization for Muconic Acid Biosynthesis**

**Han-Na Lee^1,3,&^, Woo-Shik Shin^2,&^, Seung-Yeul Seo^3,4,&^, Si-Sun Choi^1^, Ji-soo Song^1^, Ji-yeon Kim^1^, Ji-Hoon Park^1^, Dohoon Lee^2,5^, Sang Yong Kim^2,5^, Sang Joung Lee^3^, Gie-Taek Chun^4,*^, and Eung-Soo Kim^1,*^**

^1^Department of Biological Engineering, Inha University, Incheon 22212, Republic of Korea

^2^Green Chemistry and Materials Group, Korea Institute of Industrial Technology, Cheonan si, Chungcheongnam-do, 31056, Republic of Korea

^3^STR Biotech Co., Ltd., Bioplaza 4-3, 56, Soyanggang-ro, Chuncheon-si, Gangwon-do 24232, Republic of Korea

^4^Department of Molecular Bio-science, Kangwon National University, Chuncheon-si, Gangwon-do 24341, Republic of Korea

^5^Green Process and System Engineering Major, Korea University of Science and Technology (UST), Daejeon, 34141, Republic of Korea.

**^&^**Equal contribution

**^*^Corresponding author: Eung-Soo Kim**

*Department of Biological Engineering, Inha University, Incheon 22212, Korea*

Tel: 82-32-860-8318, Fax: 82-32-872-4046, E-mail: eungsoo@inha.ac.kr

^&^Co-corresponding author: Gie-Taek Chun

*Department of Molecular Bio-science, Kangwon National University Gangwon-do 24341, Korea*

E-mail: gtchun8547@gmail.com

**Supplementary Figure Legends**

**Supplementary Fig. 1.** Growth verification of *aroE (Ncgl1567)* gene disrupted strain on the different agar medium. *C. glutamicum* 13032 wild-type and STR001 strains were streaked on LB medium (A), BHIA medium (B), BT minimal medium (C) or BT minimal medium supplemented with 0.4 mg/L of three aromatic amino acids (D) and incubated at 30 ℃ for 2 days. WT, *C. glutamicum* 13032 wild-type; STR011, *C. glutamicum* 13032 Δ*aroE*; tyr, tyrosine; trp, tryptophan; phe, phenylalanine.

**Supplementary Fig. 2.** HPLC chromatogram of MA and intermediates produced in various *C. glutamicum* strains. Metabolites were detected at wavelengths of 250 nm.

**Supplementary Fig. 3.** MA production in the flask cultivation by adding benzoate into the CMP production medium. Two strains were grown in CMP production medium supplemented 0.5 g/L of benzoate initially. The cultures were carried out using 250 ml flasks for 3 days at 30 °C and 240 rpm.

**Supplementary Fig. 4.** Time course profiles of cell growth, agitation speed, DO concentration, pH, DCW, OD_600_, glucose, organic acids and metabolite production by the STR004 strain during the 3-L bath fermentation. The 1^st^ culture grown in GF1 medium was inoculated into the 2^nd^ growth culture. The 2^nd^ culture was inoculated into a 5-L fermenter (1 % v/v inoculums) for the production culture. Production culture was conducted in the FM1 medium (glucose 60 g/L, (NH_4_)_2_SO_4_ 13.5 g/L, yeast extract 3 g/L, casitone 3 g/L, KH_2_PO_4_ 5 g/L, MgSO_4_∙7H_2_O 1 g/L, citric acid 1.14 g/L, trace metals 1 ml/L, and thiamine hydrochloride 200 µg/L, pH 7.3)

**Supplementary Fig. 5.** Gene expression profiles of *C. glutamicum* STR003 strain at 7 h (A) and 12 h (B) in the presence of glucose. Based on the standardized transcription values (RPKM), the genes are arranged in the order of high expression and displayed the list of the top 30 genes. Grey boxes represent the genes selected for promoter.

**Supplementary Fig. 6.** Batch fermentation results based on the concentration of carbon source. Time course profiles of cell growth, agitation speed, DO concentration, pH, DCW, glucose, organic acids and metabolite production by the STR011 strain. Production cultivation in the 7-L fermenter was conducted by using SL#9 medium containing 25.84 g/L (A), 45 g/L (B) and 55 g/L (C) of glucose, respectively.

**Supplementary Fig. 7.** The 7-L fed-batch fermentation results based on the phosphate concentration in the feeding medium. Time course profiles of cell growth, agitation speed, DO concentration, pH, DCW, glucose, organic acids and metabolite production by the STR011 strain. Time course metabolite production using feeding medium containing different concentration of phosphate (1X, 0.5X, 0X) were shown in (A), (B) and (C), respectively.

(A) The feeding medium was sequentially injected at cultivation time periods of 35, 48, 49, 57 and 185 h at a rate of 0.2268 mL/min, 0.1134 mL/min, 0.1701 mL/min and 0.6804 mL/min. (B) The feeding medium was sequentially injected at cultivation time periods of 35, 42, 51, 61.5 and 66.5 h at a rate of 0.2268 mL/min, 0.4536 mL/min, 0.3402 mL/min and 0.6804 mL/min. (C) The feeding medium was sequentially injected at cultivation time periods of 35, 51, 71 and 81 h at a rate of 0.2268 mL/min, 0.3402 mL/min and 0.2268 mL/min.

**Supplementary Fig. 8.** Comparison of oxygen mass transfer coefficient (*k_L_a*) as a function of agitation speed (rpm) in a 7-L and 50-L fermenter system. Closed circle: 7-L fermenter; Closed triangle: 50-L fermenter.

**Supplementary Fig. 9.** Production of MA by *C. glutamicum* STR003 harboring various RBS sequences. The cultivations were conducted in 250 ml flasks for 3days at 30°C and 240 rpm in CMP production medium. RBS sequences (the spacer sequences between AAAGG and ATG initiation codon) were designed based on the results of Zhang, B. *et al* (ref 26) and cloned into the pMESK109 plasmid after fusion PCR with individual primers. Error bars represent standard deviations based on triplicate experiments.

**Supplementary Table Legends**

**Supplementary Table 1.** Primer lists used in this study.

**Supplementary Table 2.** MA production was tested by adding benzoate as sole carbon source. Two strains (WT and STR003) were grown in CMP production medium supplemented from 0 (g/L) to 1.5 (g/L) of benzoate initially. The cultures were carried out using 250 ml flasks for 3 days at 30 °C and 240 rpm. Benzoate was only accumulated in STR003 strain. Data represent the mean (**±**standard deviation) of three independent experiments.

**Supplementary Table 3.** A list of 31 variable nitrogen sources for one-factor at a time (OFAT) design.

**Supplementary Table 4.** Experimental matrix for Full Factorial Design (FFD).

**Supplementary Table 5**. ANOVA analysis of Full Factorial Design (FFD).

**Supplementary Table 6.** Experimental matrix of Steepest Ascent Method (SAM).

**Supplementary Fig. 1****.** Growth verification of *aroE (Ncgl1567)* gene disrupted strain on the different agar medium. *C. glutamicum* 13032 wild-type strain and STR001 were streaked on LB medium (A), BHIA medium (B), BT minimal medium (C) or BT minimal medium supplemented with 0.4 mg/L of three aromatic amino acids (D) and incubated at 30 ℃ for 2 days. WT, *C. glutamicum* 13032 wild-type; STR011, *C. glutamicum* 13032 Δ*aroE*; tyr, tyrosine; trp, tryptophan; phe, phenylalanine.


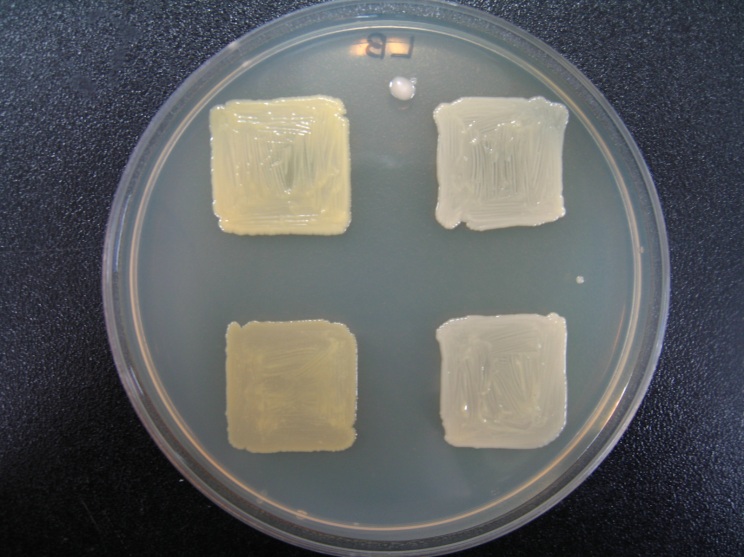


**WT**

**STR001**


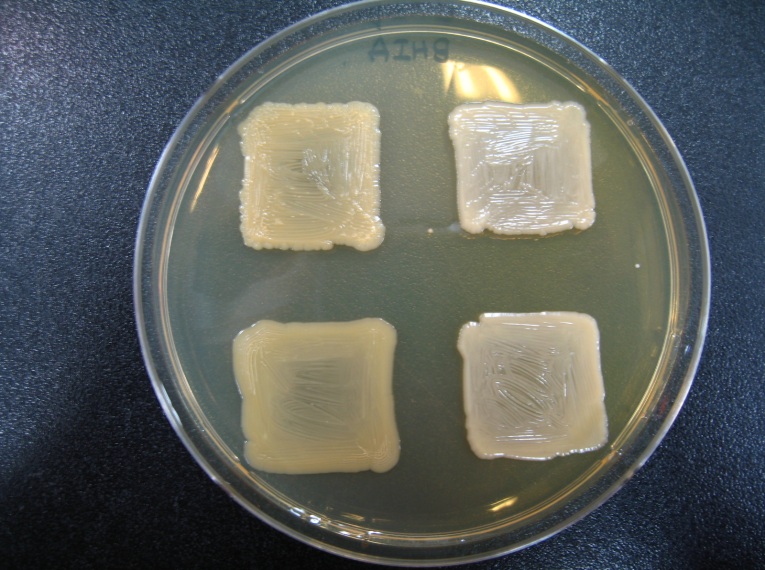


**WT**

**STR001**


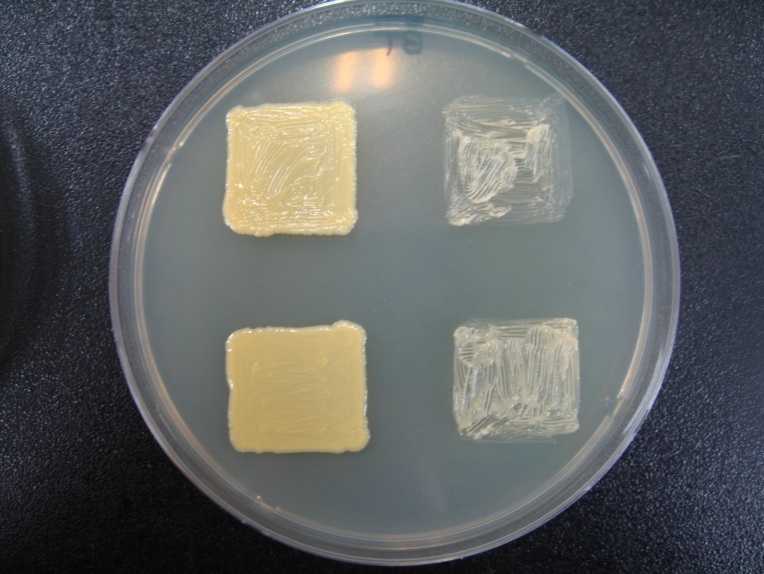


**WT**

**STR001**


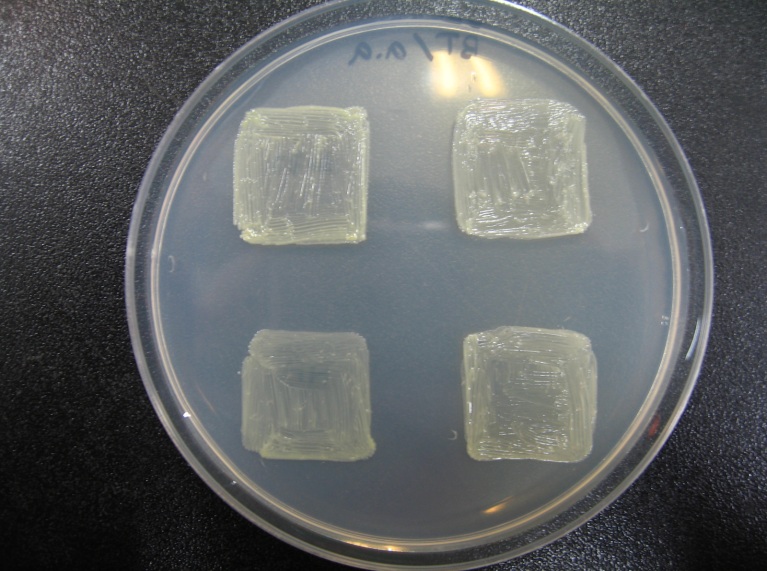


**WT**

**STR001**

**(A) LB**

**(B) BHIA**

**(C) BT**

**(D) BT / tyr, trp, phe**

**Supplementary Fig. 2.** HPLC chromatogram of MA and intermediates produced in various *C. glutamicum* strains. Metabolites were detected at wavelengths of 250 nm.


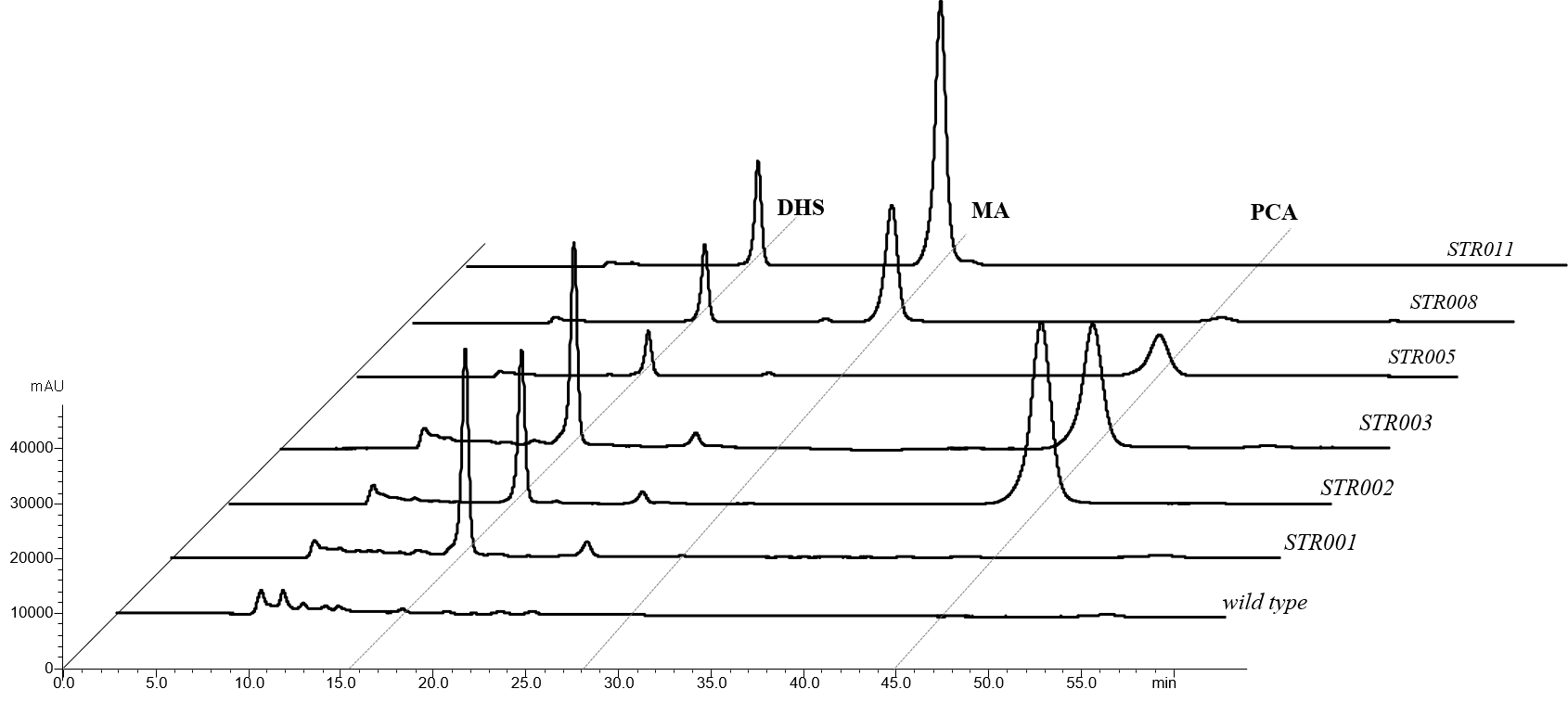


**Supplementary Fig. 3.** MA production in the flask cultivation by adding benzoate into the CMP production medium. Two strains were grown in CMP production medium supplemented 0.5 g/L of benzoate initially. The cultures were carried out using 250 ml flasks for 3 days at 30 °C and 240 rpm.

**Supplementary Fig. 4.** Time course profiles of cell growth, agitation speed, DO concentration, pH, DCW, OD_600_, glucose, organic acids and metabolite production by the STR004 strain during the 3-L batch fermentation. The 1^st^ culture grown in GF1 medium was inoculated into the 2^nd^ growth culture. The 2^nd^ culture was inoculated into a 7-L fermenter (1 % v/v inoculums) for the production culture. Production culture was conducted in the FM1 medium (glucose 60 g/L, (NH_4_)_2_SO_4_ 13.5 g/L, yeast extract 3 g/L, casitone 3 g/L, KH_2_PO_4_ 5 g/L, MgSO_4_∙7H_2_O 1 g/L, citric acid 1.14 g/L, trace metals 1 ml/L, and thiamine hydrochloride 200 µg/L, pH 7.3)

**Supplementary Fig. 5.** Gene expression profiles of *C. glutamicum* STR003 strain at 7 h (A) and 12 h (B) in the presence of glucose. Based on the standardized transcription values (RPKM), the genes are arranged in the order of high expression and displayed the list of the top 30 genes. Grey boxes represent the gene selected for promoter.


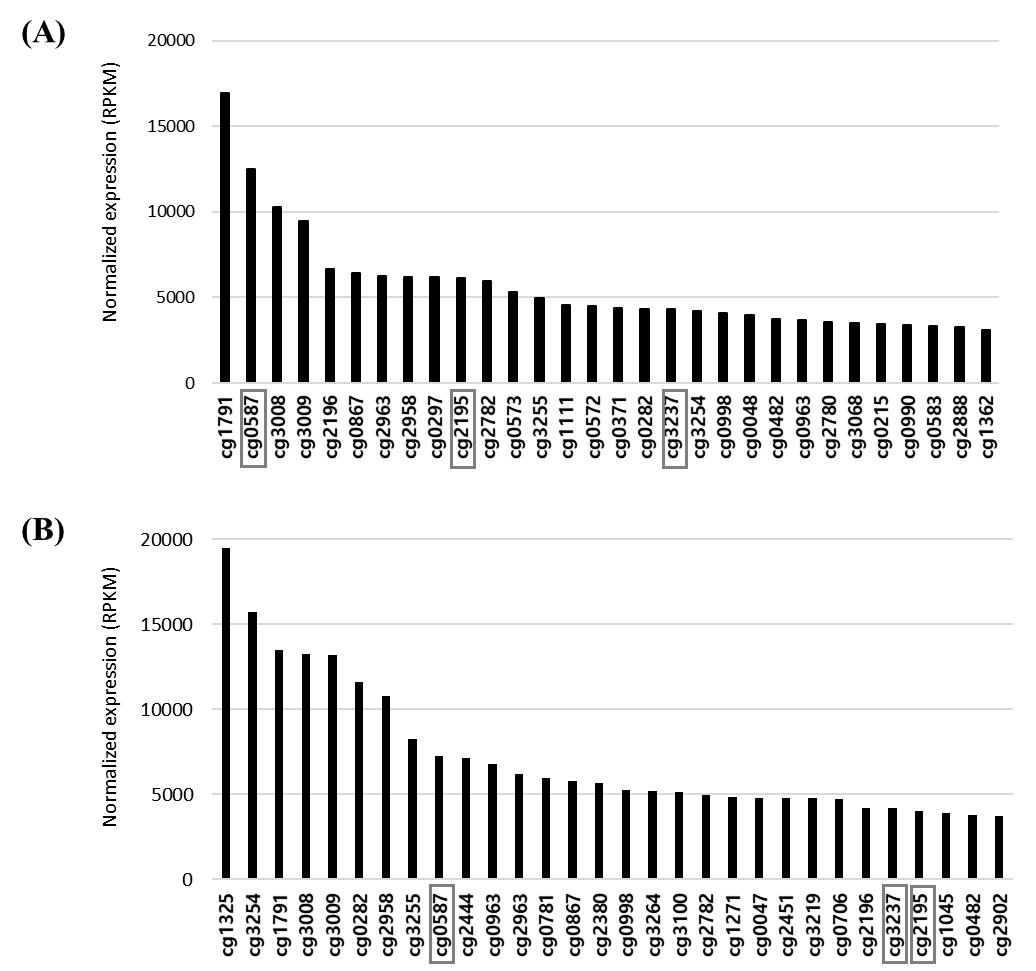


**Supplementary Fig. 6.** Batch fermentation results based on the concentration of carbon source. Time course profiles of cell growth, agitation speed, DO concentration, pH, DCW, glucose, organic acids and metabolite production by the STR011 strain. Production cultivation in the 7-L fermenter was conducted by using SL#9 medium containing 25.84 g/L (A), 45 g/L (B) and 55 g/L (C) of glucose, respectively.

(A) (B)

(C)

**Supplementary Fig. 7.** The 7-L fed-batch fermentation results based on the phosphate concentration in the feeding medium. Time course profiles of cell growth, agitation speed, DO concentration, pH, DCW, glucose, organic acids and metabolite production by the STR011 strain. Time course metabolite production using feeding medium containing different concentration of phosphate (1X, 0.5X, 0X) were shown in (A), (B) and (C), respectively.

(A) The feeding medium was sequentially injected at cultivation time periods of 35, 48, 49, 57 and 185 h at a rate of 0.2268 mL/min, 0.1134 mL/min, 0.1701 mL/min and 0.6804 mL/min. (B) The feeding medium was sequentially injected at cultivation time periods of 35, 42, 51, 61.5 and 66.5 h at a rate of 0.2268 mL/min, 0.4536 mL/min, 0.3402 mL/min and 0.6804 mL/min. (C) The feeding medium was sequentially injected at cultivation time periods of 35, 51, 71 and 81 h at a rate of 0.2268 mL/min, 0.3402 mL/min and 0.2268 mL/min.

(A) (B)

(C)

**Supplementary Fig. 8.** Comparison of oxygen mass transfer coefficient (*k_L_a*) as a function of agitation speed (rpm) in a 7-L and 50-L fermenter system. Closed circle: 7-L fermenter; Closed triangle: 50-L fermenter.

**Supplementary Fig. 9.** Production of MA by *C. glutamicum* STR003 harboring various RBS sequences. The cultivations were conducted in 250 ml flasks for 3days at 30°C and 240 rpm in CMP production medium. RBS sequences (the spacer sequences between AAAGG and ATG initiation codon) were designed based on the results of Zhang, B. *et al* (ref 26) and cloned into the pMESK109 plasmid after fusion PCR with individual primers. Error bars represent standard deviations based on triplicate experiments.

**Supplementary Table 1.** Primer lists used in this study.

| **Primer name** | **Sequence (5’🡪 3’)** | **Target** |
| --- | --- | --- |
| aroE_up_F | CTGCAGCGCTTGATGCTTTAGGTGTT | *aroE* gene disruption |
| aroE_up_R | GTCGACCGTGAGGCTTTGGAAGAGT |  |
| aroE_down_F | GTCGACAGGACTGGGGATTTGGAAT |  |
| aroE_down_R | GAATTCACCTGGCCGATATTGATCGG |  |
| aroE_con_F | acatcatcctccttgaccaaggacgtgt | *C. glutmicum* STR001 |
| aroE_con_R | gttccatcggtgtcgatggtgacaaagt |  |
| pca_up_F | GACCATGATTACGCCAAGCTTGGAATATCACCTCACGAGG | *pcaGH* gene disruption |
| pca_up_R | GGTACCCGGGGATCCTCTAGAATTGACCCGATCTTTATACTC |  |
| pca_dw_F | CGACTCTAGAGGATCCCCGGGGCGAGACCTTTCTGCGTC |  |
| pca_dw_R | AAAACGACGGCCAGTGAATTCCTAACCGCTCTACCTGCA |  |
| pca_con_F | TCCACATCCAACTGATAAGAATCAAT | *C. glutmicum* STR002 |
| pca_con_R | TCTGCGCGCATCATCACAATATGAAGA |  |
| catB LF | gccaagctttttagtcatccaatacctcga | *catB* gene disruption |
| catB LR | acagcatcgataattctcaagcttgcatgcc |  |
| catB RF | cgactctagatttttctatggctttctggtta |  |
| catB RR | cggcacactcaactgcctgatctagaggat |  |
| catB_con_F | ATCACATCGGCGTCCATGGAATCA | *C. glutmicum* STR003 |
| catB_con_R | TAGCATTGAAGTCCCGACCACATC |  |
| pMESK101_F | TTTCTACAAgAtCTTCCTGTCGTCATATCT | pSK1Cat construction |
| pMESK101_R | TCGAGATCCaCTAGATTTAAATTAATTAAGA |  |
| Ptuf_F | AAGGCTAAGGTTCCACTaGTCTCAGATGTT | Promoter cloning for pMESK106, 107 |
| Ptuf_R | CTTCGCCTTTGCCAtaTGTATGTCCTCCTGG |  |
| Psod_F | AACACACCTCGCGAcTAGTGGGATTAGGG | Promoter cloning for pMESK108, 109 |
| Psod_R | GTTCGTATACAGCatATGGGTAAAAAATCCT |  |
| P180_F | acgtcaagttaactagttaataaaggtg | Promoter cloning for pMESK104, 105 |
| P180_R | acagcaagtcatatgaatgaatttagattc |  |

**Supplementary Table 2.** MA production was tested by adding benzoate as sole carbon source. Two strains (WT and STR003) were grown in CMP production medium supplemented from 0 (g/L) to 1.5 (g/L) of benzoate initially. The cultures were carried out using 250 ml flasks for 3 days at 30 °C and 240 rpm. Benzoate was only accumulated in STR003 strain. Data represent the mean (**±**standard deviation) of three independent experiments.

|  | WT | | | | STR003 | | | |
| --- | --- | --- | --- | --- | --- | --- | --- | --- |
|  | Benzoate (g/L) | | | | Benzoate (g/L) | | | |
|  | 0 | 0.5 | 1.0 | 1.5 | 0 | 0.5 | 1.0 | 1.5 |
| MA | 0 | 0 | 0 | 0 | 0 | 0.1±0.01 | 0.12±0.02 | 0.03±0.02 |
| CA | 0 | 0 | 0 | 0.4±0.01 | 0 | 0 | 0 | 0 |
| PCA | 0 | 0 | 0 | 0 | 0 | 0 | 0 | 0 |

**Supplementary Table 3.** A list of 31 variable nitrogen sources for one-factor at a time (OFAT) design.

| **Nitrogen source** | | | |
| --- | --- | --- | --- |
| 1 | Ammonium chloride | 17 | Peptonized milk |
| 2 | Ammonium nitrate | 18 | Peptone (N-Z-soy BL7) |
| 3 | Ammonium phosphate(mono) | 19 | Peptone(N-Z-soypeptone) |
| 4 | Ammonium phosphate(di) | 20 | Peptone (N-Z-soy BL4) |
| 5 | Ammonium tartrate | 21 | Bacto peptone |
| 6 | Casamino acid | 22 | Peptone water |
| 7 | Casitone | 23 | Soytone peptone |
| 8 | Casein enzyme hydrolysate | 24 | Soytone |
| 9 | Casein acid hydrolysate | 25 | Soytone meal |
| 10 | Ammonium sulfate | 26 | Soytone flour |
| 11 | Corn steep liquor | 27 | Skim milk |
| 12 | Corn steep solid | 28 | Tryptone |
| 13 | Yeast extract | 29 | Trypticase peptone |
| 14 | Cotton seed flour | 30 | Biosate peptone |
| 15 | Cotton seed enzymatic hydrolysate | 31 | Urea |
| 16 | Glutamic acid |  |  |

**Supplementary Table 4.** Experimental matrix for full factorial design (FFD).

| **Factors** |  | **Cord levels** |  |
| --- | --- | --- | --- |
|  | **-1** | **0** | **1** |
|  | **g/L** | | |
| **Glucose** | 30 | 40 | 50 |
| **(NH_4_)_2_SO_4_** | 3.5 | 5 | 6.5 |
| **Yeast extract** | 1.25 | 2.5 | 3.75 |
| **Corn steep solid** | 1.5 | 3.5 | 5.5 |
| **KH_2_PO_4_** | 3.75 | 5 | 6.25 |

**Supplementary Table 5**. ANOVA analysis of Full Factorial Design (FFD).

|  | **Sum of** |  | **Mean** | **F** |  |  |
| --- | --- | --- | --- | --- | --- | --- |
| **Source** | **Squares** | **DF** | **Square** | **Value** | **Prob > F** |  |
| Model | 20.05341 | 15 | 1.336894 | 14.56319 | < 0.0001 | significant |
| A | 0.433561 | 1 | 0.433561 | 4.722907 | 0.0414 |  |
| B | 0.035491 | 1 | 0.035491 | 0.386617 | 0.5408 |  |
| C | 17.80346 | 1 | 17.80346 | 193.9385 | < 0.0001 |  |
| D | 0.818304 | 1 | 0.818304 | 8.914034 | 0.0070 |  |
| E | 0.076419 | 1 | 0.076419 | 0.832455 | 0.3719 |  |
| AB | 0.107672 | 1 | 0.107672 | 1.172905 | 0.2911 |  |
| AC | 0.10857 | 1 | 0.10857 | 1.182682 | 0.2891 |  |
| AD | 0.022073 | 1 | 0.022073 | 0.240449 | 0.6290 |  |
| AE | 0.000151 | 1 | 0.000151 | 0.001642 | 0.9681 |  |
| BC | 0.002517 | 1 | 0.002517 | 0.027414 | 0.8701 |  |
| BD | 0.006874 | 1 | 0.006874 | 0.074875 | 0.7870 |  |
| BE | 0.032479 | 1 | 0.032479 | 0.353804 | 0.5583 |  |
| CD | 0.009422 | 1 | 0.009422 | 0.102638 | 0.7519 |  |
| CE | 0.587915 | 1 | 0.587915 | 6.404334 | 0.0194 |  |
| DE | 0.008506 | 1 | 0.008506 | 0.092663 | 0.7638 |  |
| Curvature | 1.095469 | 1 | 1.095469 | 11.93327 | 0.0024 | significant |
| Residual | 1.92779 | 21 | 0.0918 |  |  |  |
| Lack of Fit | 1.432096 | 16 | 0.089506 | 0.902836 | 0.6052 | not significant |
| Pure Error | 0.495693 | 5 | 0.099139 |  |  |  |
| Cor Total | 23.07667 | 37 |  |  |  |  |

**Supplementary Table 6.** Experimental matrix of Steepest Ascent Method (SAM).

| **Run** | **Factors** | | | | |
| --- | --- | --- | --- | --- | --- |
|  | **Glucose** | **(NH_4_)_2_SO_4_** | **Yeast extract** | **CSS** | **KH_2_PO_4_** |
| 1 | 40 | 5 | 2.5 | 3.5 | 5 |
| 2 | 38.43 | 4.93 | 3.76 | 3.07 | 4.92 |
| 3 | 36.85 | 4.86 | 5.02 | 2.64 | 4.83 |
| 4 | 35.28 | 4.8 | 6.28 | 2.2 | 4.75 |
| 5 | 33.71 | 4.73 | 7.54 | 1.77 | 4.67 |
| 6 | 32.14 | 4.66 | 8.8 | 1.34 | 4.59 |
| 7 | 30.56 | 4.59 | 10.06 | 0.91 | 4.5 |
| 8 | 28.99 | 4.53 | 11.32 | 0.47 | 4.42 |
| 9 | 27.42 | 4.46 | 12.58 | 0.04 | 4.34 |
| 10 | 25.84 | 4.39 | 13.84 | 0 | 4.26 |
| 11 | 24.27 | 4.32 | 15.1 | 0 | 4.17 |
